# Nortopsentins as Leads from Marine Organisms for Anticancer and Anti-Inflammatory Agent Development

**DOI:** 10.3390/molecules28186450

**Published:** 2023-09-05

**Authors:** Camilla Pecoraro, Francesca Terrana, Giovanna Panzeca, Barbara Parrino, Stella Cascioferro, Patrizia Diana, Elisa Giovannetti, Daniela Carbone

**Affiliations:** 1 Department of Biological, Chemical and Pharmaceutical Sciences and Technologies (STEBICEF), University of Palermo, Via Archirafi 32, 90123 Palermo, PA, Italy; camilla.pecoraro@unipa.it (C.P.); francesca.terrana01@unipa.it (F.T.); giovanna.panzeca@unipa.it (G.P.); barbara.parrino@unipa.it (B.P.); stellamaria.cascioferro@unipa.it (S.C.); daniela.carbone@unipa.it (D.C.); 2 Department of Medical Oncology, Cancer Center Amsterdam, Amsterdam UMC, VU University Medical Center (VUmc), De Boelelaan 1117, 1081 HV Amsterdam, The Netherlands; 3 Cancer Pharmacology Lab, Fondazione Pisana per la Scienza, Via Ferruccio Giovannini 13, 56017 San Giuliano Terme, PI, Italy

**Keywords:** natural products, marine alkaloids, nortopsentin derivatives, anticancer agents, anti-inflammatory agents

## Abstract

The marine environment is an excellent source of molecules that have a wide structural diversity and a variety of biological activities. Many marine natural products (MNPs) have been established as leads for anticancer drug discovery. Most of these compounds are alkaloids, including several chemical subclasses. In this review, we focus on the bis-indolyl alkaloid Nortopsentins and their derivatives with antiproliferative properties. Nortopsentins A–C were found to exhibit *in vitro* cytotoxicity against the P388 murine leukaemia cell line. Their structural manipulation provided a wide range of derivatives with significant anti-tumour activity against human cell lines derived from different cancer types (bladder, colon, gastric, CNS, liver, lung, breast, melanoma, ovarian, pancreatic, prostate, pleural mesothelioma, renal, sarcoma, and uterus). *In vivo* assays on animal models also proved that Nortopsentins and related bis-indolyl compounds have potent anti-inflammatory activity. These remarks set the foundation for future investigations into the development of new Nortopsentin derivatives as new anticancer and anti-inflammatory agents.

## 1. Introduction

Cancer is one of the scariest diseases in the human population, especially in developing countries, and the second leading cause of death worldwide [1]. Cancer cells are characterized by abnormal proliferation, unstoppable differentiation, invasion, and metastasis [2,3,4,5,6]. The inhibition of the proliferation pathways of cancer cells constitutes an effective strategy to treat this pathology. In the search for new anticancer drugs, medicinal chemists have set their sights on natural products, particularly the secondary metabolites of marine organisms, for several reasons. Firstly, living in such relatively closed surroundings characterized by high salt, high pressure, low temperature, hypoxia, and lack of light, marine organisms have developed, in their process of long-term evolution, a distinct metabolism system and immune system which are completely different from the terrestrial ones. Secondly, they have formed and accumulated large numbers of bioactive compounds with structural diversity and complexity, such as fatty acids, proteins, alkaloids, peroxides, coumarins, and terpenes [7,8,9]. Unsurprisingly, marine products tend to exhibit greater potency than terrestrial ones to be effective against predators because their release into the environment is quickly followed by a dilution by water [10,11,12,13,14,15,16].

The marine environment provides a wealth of marine invertebrates such as sponges, coelenterates, tunicates, bryozoans, red algae, acorn worms, and symbiotic bacteria. More than 300,000 species have been found in the ocean, and it is estimated that more than 1 million new species have not been found [16,17,18]. Among marine organisms, sponges have proved to be an abundant reserve of compounds with antibacterial, antiviral, anti-inflammatory, immunomodulatory, and antiproliferative activities [19,20]. The first marine-derived drug on the market was Cytosar^®^, whose active compound acting as an inhibitor of DNA polymerase, cytarabine (Ara-C), is a synthetic analogue of a C-nucleoside isolated from the Caribbean sponge *Tethya crypta*. Cytosar^®^ was approved by the Food and Drug Administration (FDA) in 1969 for the treatment of leukaemia and lymphoma (Table 1) [17,18,21]. Another drug available on the market is Halaven^®^, which was accepted by the FDA in late 2010 as a chemotherapy agent for the treatment of metastatic breast cancer. Its active compound, Eribulin, is an antimitotic agent and an analogue of Halichondrin-B, a macrocyclic polyether initially extracted from *Halichondria okadai* sponge in 1986. It causes G2/M cell cycle arrest by acting on tubulin or microtubules [22,23,24].

Many marine natural products have been shown to have a good inhibitory effect on human cancer cell lines during activity screening [16,20,25], in particular alkaloids [16,26,27]. Besides antiproliferative activity, marine alkaloids possess a high degree of biological activities, such as anti-inflammatory, antiviral, antimalarial, anti-fungal, antibacterial, antiosteoporosis, and immunomodulatory activity [16,22,28,29,30,31,32,33,34,35]. Alkaloids can be classified in terms of their chemical structure into subclasses such as sterols, pyridoacridines, pyrazinones, pyrroles, isoquinolines, guadinines, aminoimidazoles, indoles, and bis-indoles [36]. In particular, bis-indolyl alkaloids and their synthetic derivatives, consisting of two indole moieties linked to each other via heterocyclic units or linear chains, are well known because of their broad spectrum of biological properties, including anti-microbial [37,38], anti-viral [39,40], and anti-tumour activities [41,42,43,44,45,46,47,48,49]. Herein, the anticancer activities of Nortopsentins and their derivatives are reviewed.

## 2. Results and Discussion

**Figure 1:**
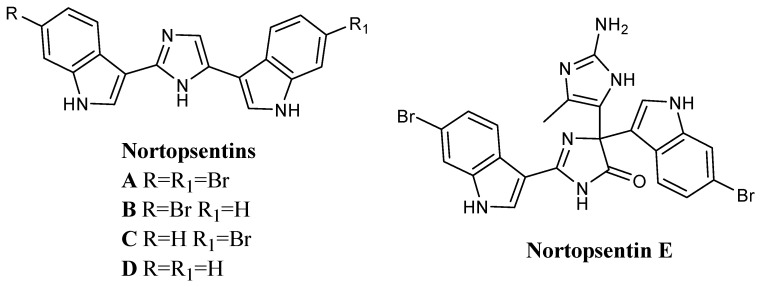
Nortopsentins.

Among marine bis-indolyl alkaloids, Nortopsentins (Figure 1) represent promising lead compounds that have attracted remarkable attention due to their *in vitro* cytotoxicity. Many Nortopsentin analogues have been synthesised and showed interesting biological activities such as cytotoxic, anti-inflammatory, antiplasmodial, antibacterial, antifungal, and insecticidal [20,50,51,52,53].

Nortopsentins A–C, isolated from the Caribbean deep-sea sponge *Spongosorites ruetzleri* in 1988, have a characteristic 2,4-bis-indolyl-imidazole skeleton and showed significant antiproliferative and antifungal activity [52]. They exhibited *in vitro* cytotoxicity against the P388 murine leukaemia cell line (IC_50_ values of 7.6, 7.8, and 1.7 µM, respectively), antifungal activity against *Candida albicans* (MIC values of 3.1, 6.2, and 12.5 µM, respectively) [52] and anti-microbial activity against *Bacillus subtilis* [54,55]. In addition, tri- and tetramethylated derivatives of Nortopsentin B exhibited remarkable improvement in *in vitro* cytotoxicity against the P388 cells when compared to the activity of the parent compound (IC_50_ values of 0.9 and 0.34 μM, respectively) [52].

Catalytic hydrogenation of Nortopsentin A–C yielded the synthetic analogue D (sometimes referred to in the literature as Nortopsentin D).

In 1996, a new bis-indolyl alkaloid was isolated, Nortopsentin E [53,55,56]. Nortopsentin E was originally isolated from the axinellid sponge *Dragmacidon* in deep waters south of New Caledonia and later from the sponge *Agelas dendromorpha*. It is a structural variant of the Nortopsentin family bearing a complex central trisubstituted (4*H*)-imidazol-4-one with a 6-bromoindole at the C2 position and a 4-methyl-1*H*-imidazol-2-amine and 6-bromoindole at C5, whose total synthesis has recently been reported [53]. Surprisingly, while Nortopsentin E was inactive on KB tumour cells *in vitro*, its methylated derivative showed both high cytotoxicity on KB cell lines (IC_50_ 0.014 µM) and antifungal activity against yeast [53,55,56].

### 2.1. Nortopsentin Derivatives as Antiproliferative Agents

#### 2.1.1. Thiazoles

**Figure 2:**
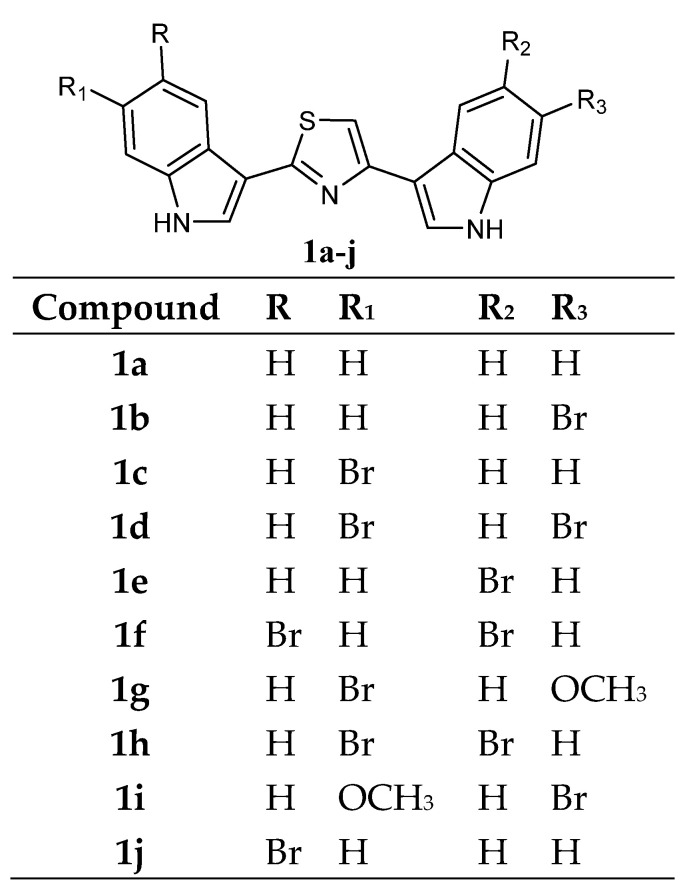
Bis-indolyl-thiazole compounds **1**.

Several bis-indolyl-thiazole compounds **1** (Figure 2) were synthesised and tested against the National Cancer Institute (NCI, Bethesda, MD 20892, USA) full panel of 60 human cancer cell lines derived from nine cancer cell types and grouped into disease subpanels including leukaemia, non-small cell lung, colon, central nervous system, melanoma, ovarian, renal, prostate, and breast cancers. Many compounds showed GI_50_ values in the micromolar-submicromolar range. In particular, compounds **1a**–**j** exhibited cytotoxic activities against a variety of human cancer cell lines. The compound **1a** exhibited highly selective *in vitro* cytotoxicity against leukaemia (GI_50_ of 3.27 µM in K562, 5.31 µM in Molt-4) and ovarian cancer cell lines (GI_50_ 8.14 µM in IGROV1) (Table 1). In many other human tumour cell lines, the GI_50_ of compound **1a** exceeded 100 µM. It is worth noting that unlike the unsubstituted compound **1a**, the bis-indolyl-thiazoles **1b**–**j** showed broad effects on leukaemia, colon, CNS, and breast cancer panels, suggesting that substituents in the indole ring might result in a potency increase (Table 2) [57,58].

**Figure 3:**
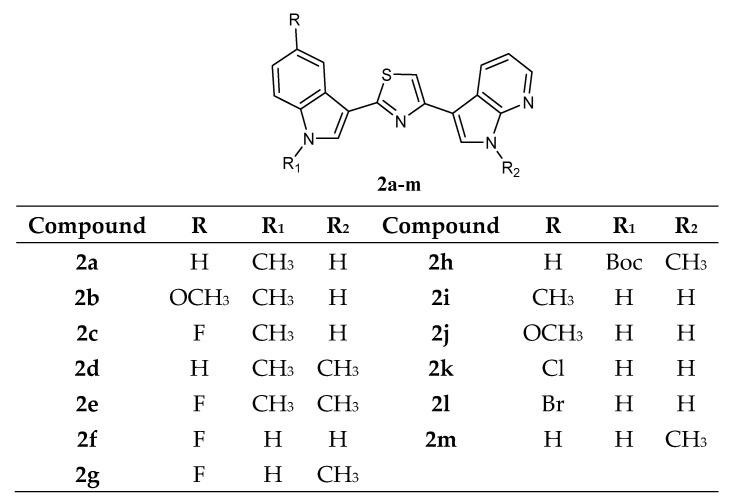
Thiazole series **2a**–**m**.

Another thiazole series **2a**–**m** (Figure 3), bearing an indole and a 7-azaindole moiety, has been reported. These derivatives were tested by NCI against a panel of 60 human cancer cell lines.

Data revealed that these compounds **2a**–**m** showed GI_50_ values in the micromolar–submicromolar range. The five most active compounds, **2c**, **2d**, **2e**, **2g**, and **2m**, which did not show selectivity against any of the tumour subpanels, were further investigated in two additional cell lines, STO and MesoII, derived from human diffuse malignant peritoneal mesothelioma (DMPM), a tumour type not included in the NCI panel. Seventy-two hours of exposure to increasing concentrations of each compound resulted in dose-dependent cell proliferation inhibition in both cellular models. Compounds **2c**, **2d**, **2e**, **2g**, and **2m**, exhibited comparable activity in STO cells with IC_50_ values ranging from 0.33 to 0.61 μM. By contrast, a variable growth inhibitory effect was induced by the different compounds in MesoII cells (IC_50_ values ranging from 4.11 to 25.12 μM). In addition, compounds **2c**, **2e**, and **2m**, did not interfere with the growth of normal cells (Table 3). The anti-tumour activity of **2c**, **2e**, and **2m** derivatives was then evaluated on STO cells xenotransplanted in athymic nude mice. The treatment with the different compounds resulted in marked tumour growth inhibition. Specifically, at the end of the experiment, a statistically significant tumour volume inhibition (TVI) compared with the control (73%, 75%, and 58%, for **2c**, **2e**, and **2m** derivatives, respectively) was observed, and two complete responses (disappearance of tumour) were also identified in each treatment group (Table 4). Moreover, the compounds **2c**, **2e**, and **2m** were well tolerated without any appreciable sign of toxicity. *In vitro* kinase assays revealed CDK1 inhibition exerted by the compounds with IC_50_ values of 0.89, 0.75, and 0.86 μM, respectively, for the derivatives **2c**, **2e**, and **2m** (Table 5). These results were comparable to those reported for two well-known CDK1 inhibitors, roscovitine and purvanalol A. In addition, derivatives **2c**, **2e**, and **2m** were able to inhibit GSK3β, but only at higher concentrations (IC_50_ values of 42.18, 40.18, and 35.68 μM, respectively) (Table 5). Further investigations revealed a marked time-dependent cell cycle arrest at the G2/M phase and an increase in the apoptotic rate by reducing the phosphorylated form of the antiapoptotic protein survivin. Moreover, the addition of compound **2m** to paclitaxel-treated cells resulted in a synergistic cytotoxic effect due to an increased apoptotic response [20].

**Figure 4:**
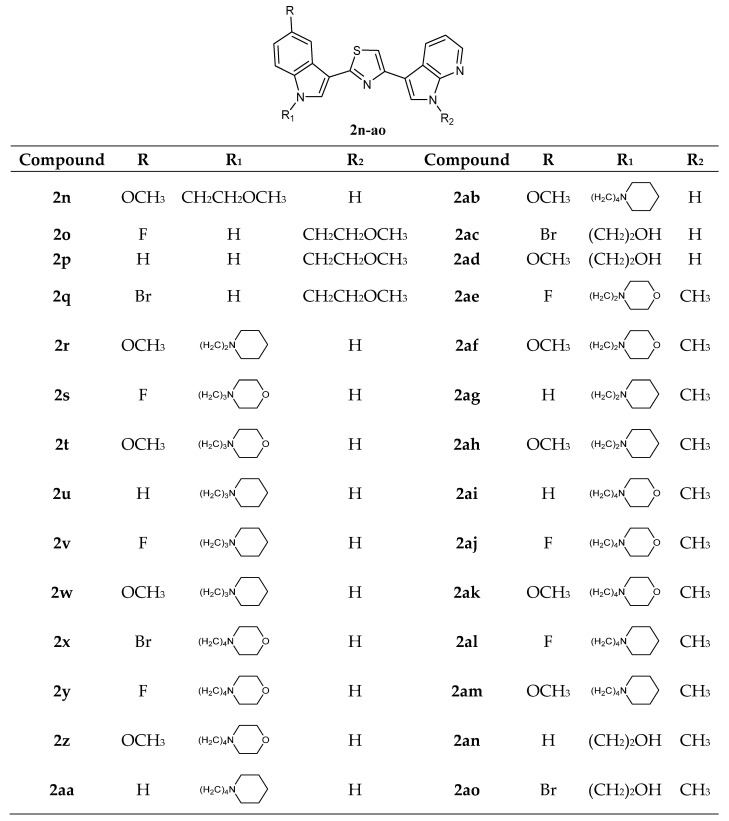
Thiazole series **2n**–**ao**.

Thiazole Nortopsentin analogues of type **2n**–**q** (Figure 4), in which the nitrogen atom of the indole and/or 7-azaindole moiety is substituted with a 2-methoxyethyl chain, and analogues **2r**–**2ao**, in which indole nitrogen is substituted with alkylmorpholine or alkylpiperidine, were synthesized. Derivatives **2n**–**2q**, **2t**–**w**, **2z**, **2ab**, **2ae**, and **2ak**–**am** were tested by NCI on the full panel of approximately 60 human cancer cell lines and showed good antiproliferative activity with GI_50_ in the micromolar–nanomolar range. Compounds **2n**, **2q**, **2t**–**w**, **2z**, **2ab**, **2ae**, and **2ak**–**am** were active against the total number of cell lines investigated, whereas compounds **2o** and **2p** were cytotoxic against a very high percentage of the tested cell lines (96% and 93%, respectively). Their action mechanism, investigated on human breast cancer MCF-7 cells, was pro-apoptotic, being associated with externalisation of plasma membrane phosphatidylserine and DNA fragmentation, accompanied by perturbation of the cell cycle progression. It was found that the derivatives **2n**–**p** confined viable cells in the G2/M phase. Derivative **2n** showed the most interesting *in vitro* anticancer activity, expressing lower GI_50_ values (0.03–12.6 µM) and, markedly, *in vitro* inhibited CDK1 activity with an IC_50_ value of 1.14 ± 0.09 µM, comparable to that reported for other indolyl-thiazolyl-7-azaindole derivatives or well-known CDK1 inhibitors, roscovitine and purvanalol A. Moreover, cytotoxicity assays on intestinal normal-like differentiated Caco-2 cells after treatment with compounds **2n**–**q** in the 25–100 µM range revealed that these compounds were selectively cytotoxic to cancer cells (Table 6) [20].

Among the derivatives **2r**–**2ao**, compound **2ak** was the most active of the series, showing selectivity against leukaemia and colon cancer subpanels. In addition, **2ak** was effective against the A498 cell line of the renal cancer subpanel (GI_50_ value of 20 nM) (Table 7). Cytotoxicity and selectivity experiments were performed for compounds **2r**–**2ao** using the HepG2 cell line of human hepatoma, the MCF-7 cell line of human breast cancer, and the non-tumorigenic MCF 10A cell line by single-dose administration (10 µM). Most of the molecules **2r**–**2ao** showed antiproliferative effects on both Hep G2 and MCF-7 cells. Some of these molecules (**2r**, **2ac**, **2aa**, **2ad**, **2ae**, **2am**, **2an**, and **2ao**) showed cytotoxicity against the tumour cell lines without compromising non-tumorigenic MCF-10A cell viability. The N-alkylpiperidine substituted compounds (**2u**, **2v**, **2w**, **2ag**, and **2ah**) were very active against the MCF-7 cell line but were also cytotoxic over MCF-10A cell line. Among the alkylmorpholino derivatives, propyl- and ethyl-morpholino derivatives (**2t** and **2ae**) were active over both Hep G2 and MCF-7 cells and non-toxic over non-tumorigenic cells, while compound **2af** was active on cancer and non-cancer cell lines examined. On the other hand, butylmorpholino derivatives gave different results, being **2s**, **2ai**, **2aj**, and **2ak** cytotoxic, while **2x** and **2y** did not impair non-tumorigenic cells; in particular, the compound **2y** showed selective toxicity on the Hep G2 cancer cell line (EC_50_ values of 3.25, 23.05, and 29.09 µM for Hep G2, MCF-7, and MCF-10A cell lines, respectively). Moreover, considering the overexpression of the enzyme glutaminase-1 (GLS-1) in hepatic cancer cell lines and its low expression in the MCF-7 cell line, enzymatic assays were also performed, revealing good inhibitory potency of the compound **2y** over GLS-1 (IC_50_ value of 3.96 µM). This could explain the selective cytotoxicity shown by **2y** on Hep G2 as opposed to MCF-7 and MCF-10A. Additional experiments on aggressive cancer cell lines with GLS-1 overexpression, like glioblastoma (U-87 MG), pancreatic cancer (MIA PaCa-2), osteosarcoma (Saos2), melanoma (A-375), and non-small lung cancer (A549) confirmed the cell growth inhibition potency for the compound **2y**, with EC_50_ values in the micromolar range (Table 8). Data suggest that the decoration of the nitrogen atom of the indole and/or 7-azaindole moiety with 2-methoxyethyl, alkylmorpholine, or alkylpiperidine chain led to interesting biological results [20,59].

**Figure 5:**
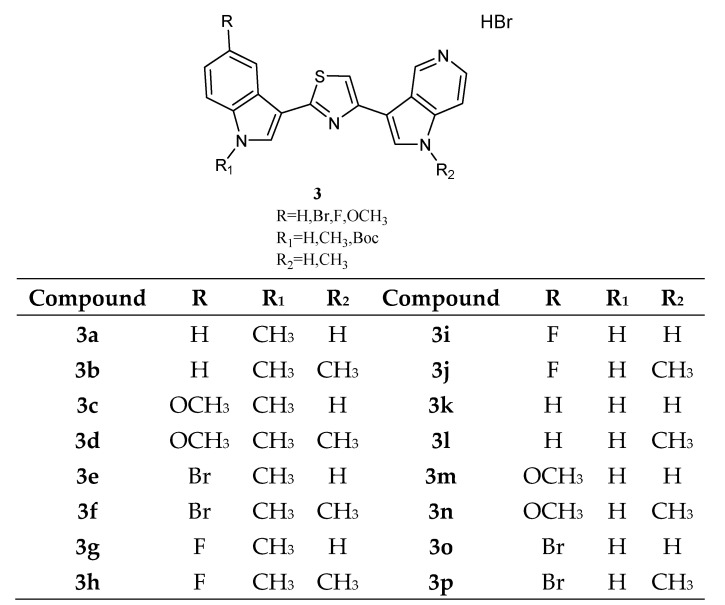
Thiazole series **3**.

A series of thiazole Nortopsentin analogues of type **3** (Figure 5), in which the imidazole moiety of Nortopsentins was replaced by a thiazole ring and one indole unit by a 5-azaindole ring, was synthesized. Derivatives **3a**–**p** were active against the NCI full panel, showing good antiproliferative activity in the micro–submicromolar range. Thiazoles **3e**, **3f**, and **3p** were particularly cytotoxic against the leukaemia subpanel (GI_50_ in the range 0.24–1.71 µM, 0.24–1.57 µM, and 0.35–2.13 µM, respectively) (Table 9 and Table 10). Compound **3b** turned out to be the most active against the breast cancer subpanel (GI_50_ in the range 0.27–2.16 µM). Moreover, compounds **3b** and **3f** proved to be selective against the HCT-116 cell line of the colon cancer subpanel (GI_50_ of 0.93 µM and 0.18 µM, respectively) [60].

**Figure 6:**
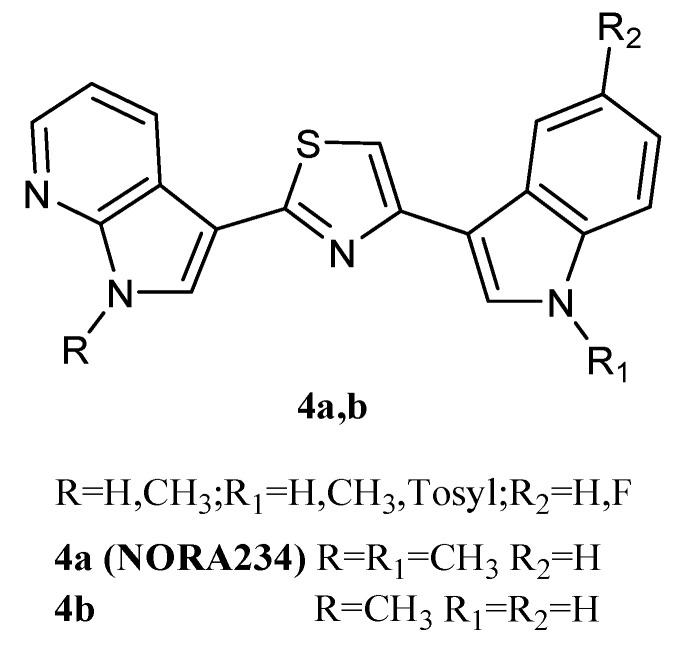
Thiazole derivatives **4a** (NORA234), **4b**.

Thiazole derivatives **4a**,**b** (Figure 6), in which indole and 7-azaindole units were switched compared to derivatives **2**, were also reported. The derivatives **4a** and **4b** showed good antiproliferative activity against the NCI full panel (about 60 human tumour cell lines) with GI_50_ values ranging from low micromolar to nanomolar levels (0.03–13.0 and 0.04–14.2 μM, respectively) (Table 11). They also exhibited potent cytotoxicity on HepG2 hepatocarcinoma cells, a cell line not included in the NCI panel. Both compounds inhibited the HepG2 cells growth in a dose-dependent manner, with GI_50_ values of 1.69 and 0.21 µM for **4a** and **4b**, respectively. Under the same conditions, both compounds did not affect the viability of normal immortalised human liver cells Chang, proving to be selective towards tumour cells. The mechanism of action of these derivatives was pro-apoptotic, being associated with the externalisation of plasma membrane phosphatidylserine and mitochondrial dysfunction. Both compounds **4a** and **4b** caused a significant dose-dependent decrease in the percentage of cells in the G0/G1 and S phases, coupled with an increase in cells in the G2/M phase, and the appearance of a subG1-cell population [20].

In addition, the compound **4a**, called Nortopsentin 234 (NORA234) (Figure 6) was found to lead to an initial reduction in the proliferative and clonogenic potential of advanced colorectal cancer sphere cells (CR-CSphCs), followed by an adaptive response selecting the CR-CSphC-resistant compartment. Cells saved from the treatment with NORA234 expressed high levels of CD44v6, combined with constitutive activation of the Wnt pathway. In CR-CSphC-based organoids, NORA234 caused genotoxic stress with concomitant G2/M cell cycle arrest and activation of CHK1, driving the DNA damage repair of CR-CSphCs, regardless of the mutational background, microsatellite stability, and consensus molecular subtype. The synergic combination of NORA234 and a CHK1 inhibitor (rabusertib) targeted synthetic lethal inducing death in both CD44v6-negative and CD44v6-positive CRC stem cell fractions, apart from Wnt pathway activity. These data could provide a rationale for developing an effective strategy for the treatment of colorectal cancer (CRC) [61].

**Figure 7:**
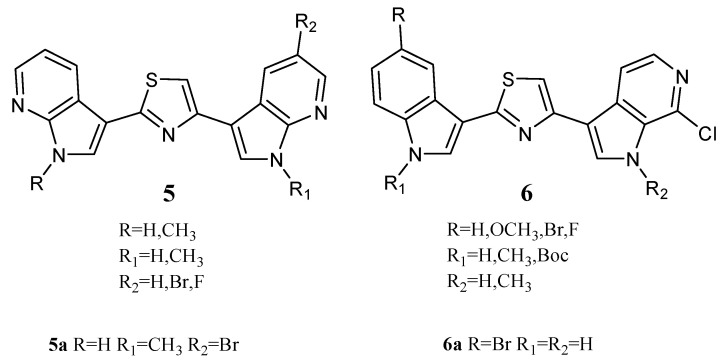
Thiazole series **5**,**6**.

Two series of Nortopsentin thiazolyl analogues **5** and **6** (Figure 7) were also synthesized. Compared to derivatives **2**, in derivatives **5**, both indole units were replaced by 7-azaindole moieties, while in derivatives **6**, one indole unit was replaced by a 6-azaindole unit. In these series, compounds **5a** and **6a** showed cytotoxic activity against a broad spectrum of human cancer cell lines included in the NCI panel, having GI_50_ values of 0.81–27.7 and 0.93–4.70 µM, respectively (Table 11). The indolyl-thiazolyl-pyrrolo[2,3-*c*]pyridine derivative **6a** resulted in more active than thiazolyl-bis-pyrrolo[2,3-*b*]pyridines derivative **5a** in terms of GI_50_. Interestingly, the compounds did not significantly compromise the vitality of intestinal normal-like differentiated Caco-2 cells, providing tumour cells as the main target of their cytotoxicity. Investigation of the mechanisms behind the antiproliferative activity in HCT-116 colon cancer cells showed that derivative **5a** caused a dose-dependent increase in the apoptotic cell population, engaging the mitochondria-mediated pathway and causing cell cycle arrest at the G2/M phase. On the other hand, derivative **6a**, at a concentration lower than its GI_50_, exhibited antiproliferative effects with a great accumulation of autophagic vacuoles without apparent signs of apoptosis. The arrest of the cell cycle at G1 phase proved the autophagic fate of the cells. Evidence indicates that autophagic cell death can be induced as an alternative to apoptosis with therapeutic finality in cancer cells that are resistant to apoptosis. It follows that thiazole compound **6a** could be considered a lead compound for Nortopsentin derivatives with autophagic activity [20].

**Figure 8:**
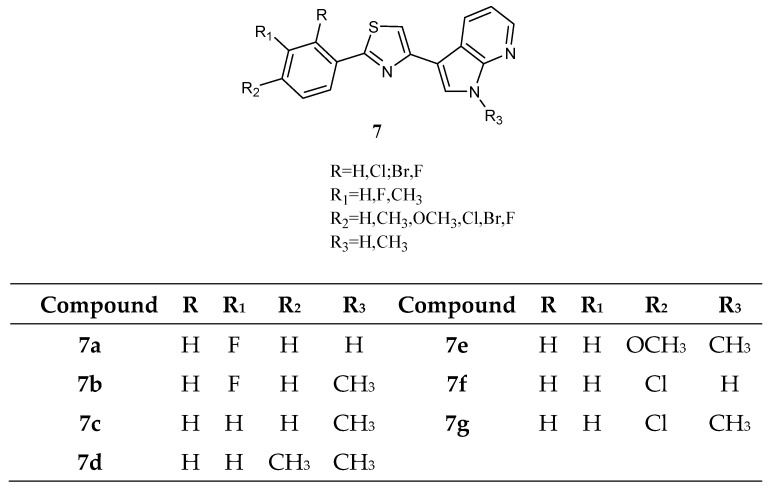
Thiazole series **7**.

Among thiazole analogues, derivatives **7** (Figure 8), in which one indole ring was replaced by a phenyl while the other one was replaced by a 7-azaindole, were also reported.

Derivatives **7b**,**c**,**d**, and **g** were active against all cancer cell lines tested by NCI, while derivatives **7a**,**e**, and **f** were cytotoxic against a good percentage of the tested cell lines (50%, 70% and 15% respectively), having GI_50_ values in the micromolar to sub-micromolar/nanomolar range (Table 11). The most active compounds were the *N*-methyl derivatives, and four of them, **7b**,**7c**,**7d**, and **7g**, were further tested against pancreatic carcinoma (MiaPaCa-2) and malignant peritoneal mesothelioma (STO) showing IC_50_ values in the range 4.3–41.6 µM and 0.41–17.2 µM, respectively (Table 12). Kinase activity assays (CDK1/cyclin, CDK5/p25, or GSK3β) were also performed to explain the mechanism of action of this series of compounds. Only the compounds with the highest antiproliferative activity (**7c** and **7d**) exhibited affinity for CDK1, with IC_50_ values of 0.41 and 0.85 µM, respectively. Such values were like those of roscovitine and purvanalol A, used as reference drugs (Table 13). Moreover, exposure of asynchronously growing STO cells to both compounds affected cell-cycle phase distribution, leading to a concentration-dependent accumulation of cells in the G2/M phase with a concomitant increase in the sub-G1 apoptotic cell population [62].

**Figure 9:**
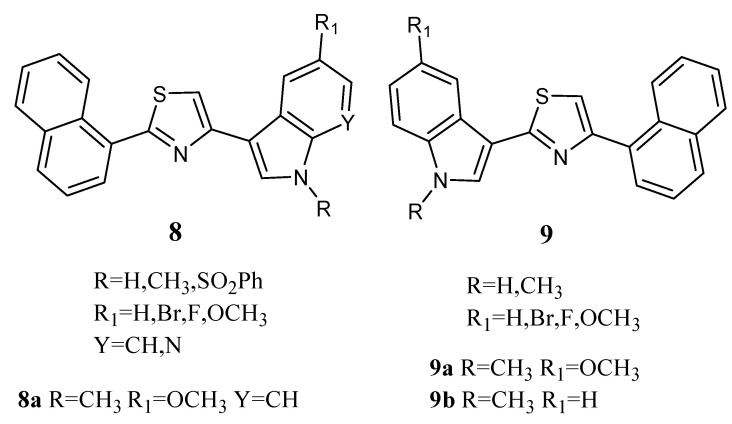
Thiazole series **8**,**9**.

Thiazole derivatives **8** and **9** (Figure 9) combine an indole unit with a naphthalyl portion. Derivatives **8a**, **9a**, and **9b** in particular displayed good antiproliferative activity against the MCF-7 cell line with GI_50_ values in the micromolar range (2.13, 3.26 and 5.14 µM, respectively, Table 14). Their mechanism of action was found to be pro-apoptotic, inducing early apoptosis in MCF-7 cells after 24 h of treatment without necrotic effects. They also caused a decrease in the percentage of cells in the G0/G1 and S phases, with a concomitant percentage increase in cells in the G2/M phase [63].

#### 2.1.2. Thiadiazoles

**Figure 10:**
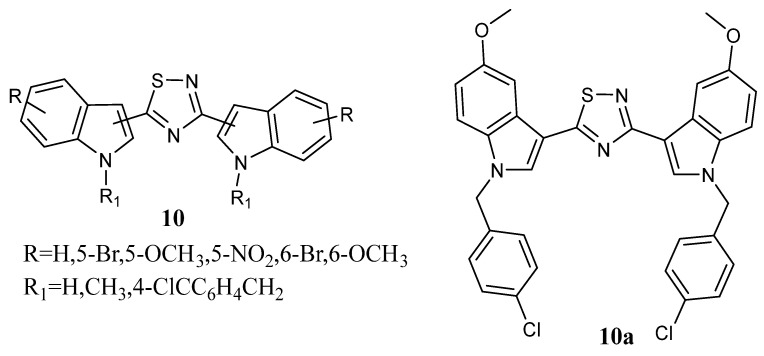
Bis-indolyl-1,2,4-thiadiazole derivatives **10**.

A bis-indolyl-1,2,4-thiadiazole series **10** (Figure 10) was screened for *in vitro* cytotoxicity against six human cancer cell lines: prostate (PC3, DU145, and LnCaP), breast (MCF-7 and MDA-MB-231), and pancreas (PaCa2). In this series, indolyl-1,2,4-thiadiazole **10a** was identified as the most potent compound with IC_50_ values of 14.6, 21.4, and 21.2 µM against the cancer cell lines LnCaP, PC3, and PaCa2, respectively (Table 15) [64].

#### 2.1.3. Thiophenes

**Figure 11:**
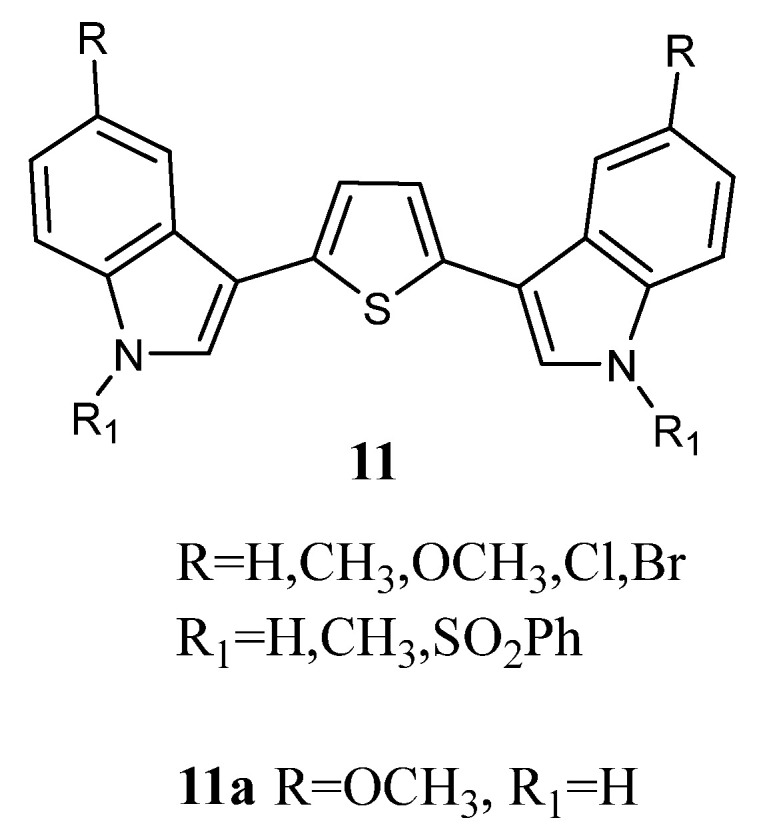
Bis-indolyl-thiophene derivatives **11**.

Bis-indolyl-thiophene derivatives of type **11** (Figure 11), in which the imidazole moiety of Nortopsentin was replaced by a thiophene ring, were submitted to the NCI for evaluation of the full panel (about 60 human cancer cell lines). The most active compound was the derivative **11a**, having GI_50_ values in the range of 0.34–19.0 µM. It was particularly powerful against the leukaemia subpanel, having GI_50_ in the range of 0.34–3.54 µM (Table 16) [63].

#### 2.1.4. Furans

**Figure 12:**
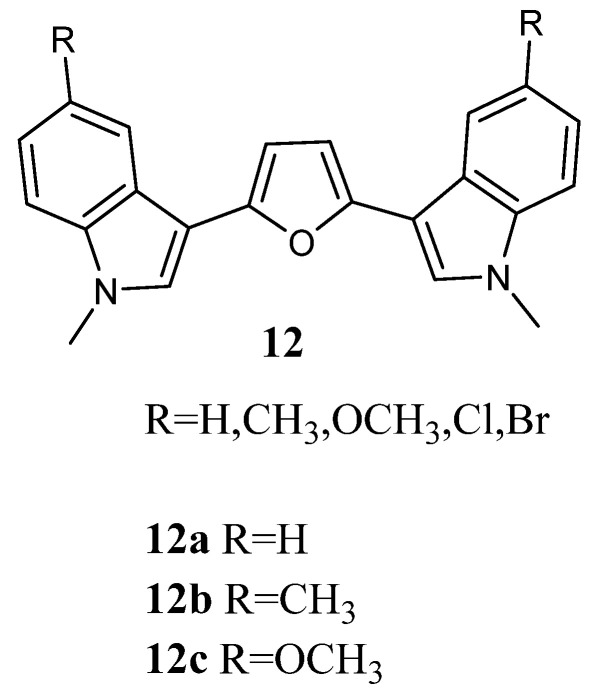
Bis-indolyl-furan derivatives **12**.

Bis-indolyl-furan derivatives **12a**–**c** (Figure 12) were screened for *in vitro* anti-tumour activity in a panel of 10 human tumour cell lines and showed mean IC_50_ values of 27.1, 21.1, and 17.1 µg/mL, respectively (Table 16). The most active candidate, **12c**, was further screened in a panel of 29 cell lines, showing cytotoxicity against a high percentage of tested cell lines (76%) with mean IC_50_ values of 20.5 µg/mL (53.1 µM). Moreover, compound **12c** was further tested by the NCI on a panel of approximately 60 tumour cell lines. Data revealed that compound **12c** was cytotoxic against all cell lines investigated, displaying GI_50_ values at micromolar concentration and was particularly potent against the leukaemia subpanel, having GI_50_ values in the range of 1.63–6.46 µM (Table 17) [63].

#### 2.1.5. Pyrroles

**Figure 13:**
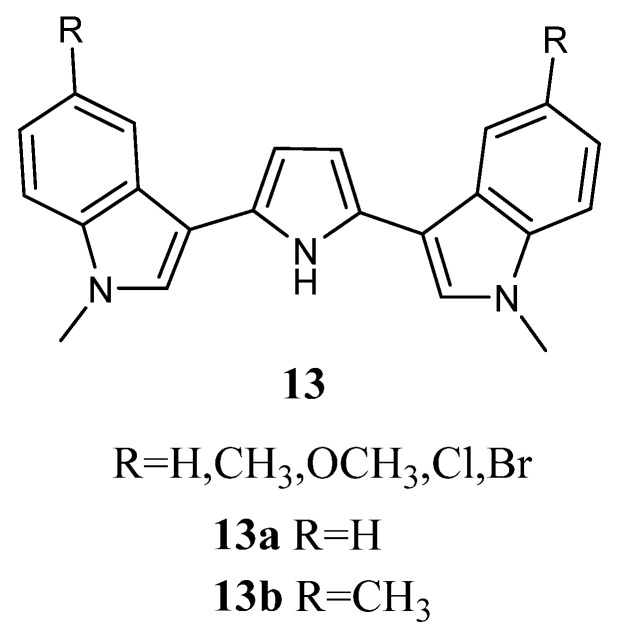
Bis-indolyl-pyrrole derivatives **13**.

Bis-indolyl-pyrroles of type **13** (Figure 13) were investigated *in vitro* against human tumour cell lines and by ex-vivo clonogenic assay using human tumour xenografts. Screening in monolayer cultures of 42 human tumour cell lines derived from 15 different solid tumour types (bladder, colon, gastric, head-neck, liver, lung, mammary, melanoma, ovarian, pancreatic, prostate, pleural mesothelioma, renal, sarcoma, and uterus) revealed that the most active derivatives were **13a** and **13b**. Compounds **13a** and **13b** affected concentration-dependent inhibition of tumour cell growth with IC_50_ values in the range 0.22–6.34 μM and 0.11–2.65 μM, respectively, indicating pronounced cytotoxic potency (Table 18). Regarding compound **13a**, selective activity was observed with submicromolar IC_50_ values against some cell lines, such as cell lines of bladder cancer (BXF 1218L, BXF 1352L), gastric cancer cell line (GXA MKN45), head-neck cell line (HNXF CAL27), two melanoma cell lines (MEXF 1341L; MEXF 276L), as well as LXFL 1121L (lung cancer), PAXF PANC-1 (pancreatic cancer), PRXF PC3M (prostate cancer), SXF SAOS-2 (sarcoma), and UXF 1138L (cancer of the uterine body) cell lines. Less sensitive cell lines were found among colon (HCT-116, HT-29), lung (LXFA 289L), ovarian (OVXF 899L), prostate (DU145), and renal cancer (RXF 393NL, RXF 486L). Compound **13b** exhibited pronounced activity with submicromolar IC_50_ values in 32 cell lines.

The anti-proliferative activity of **13a** and **13b** was also evaluated in cell suspensions prepared from 44 human tumour xenografts of 13 different tumour types (bladder, colon, gastric, head-neck, lung, mammary, melanoma, ovarian, pancreatic, prostate, pleural mesothelioma, renal, and sarcoma), which were cultured as solid tumours in serial passage on immune-deficient nude mice (Table 18). The results confirmed the concentration-dependent activity of **13a** and **13b** on cell lines with IC_50_ values in the range of 1.18–54.90 μM and 0.37–40.70 μM, respectively. Selectivity was encountered for **13a** against 9 out of the 44 tumours tested, while these sensitive tumours were scattered among various tumour histotypes, like bladder, gastric, head and neck, lung cancer, melanoma, and pleuramesothelioma. Better tumour selectivity was displayed for compound **13b**, with 14 out of 44 tumours. Sensitive cancer types were found among the bladder, head and neck, lung, pancreatic, prostate, renal cancer, melanoma, and pleuromesothelioma [16].

#### 2.1.6. Oxazoles

**Figure 14:**
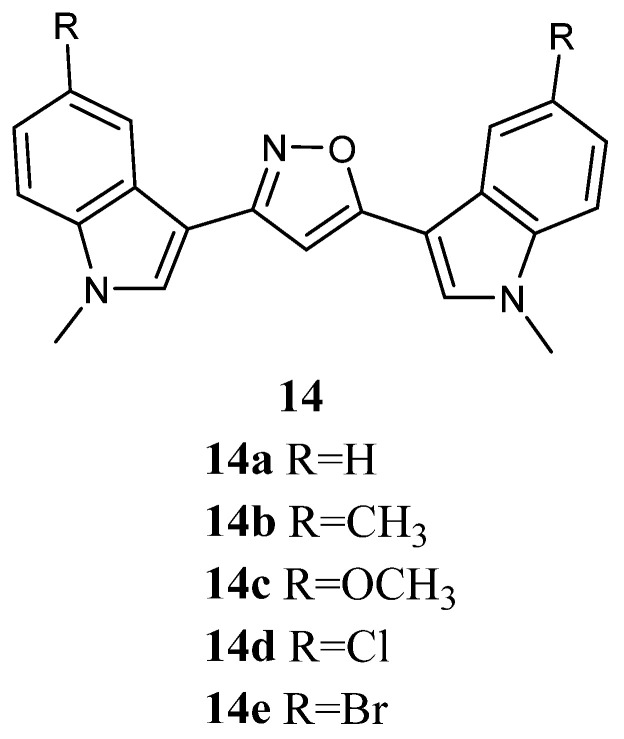
Bis-indolyl-isoxazole derivatives **14**.

Bis-indolyl-isoxazoles **14a**–**e** (Figure 14), were screened for *in vitro* anti-tumour activity in a panel of 10 human tumour cell lines by using a monolayer cell survival and proliferation assay. All compounds showed cytotoxic activity, exhibiting mean IC_50_ values in the range of 9.6–44.5 µg/mL (Table 19). Moreover, the most active candidate **14a** was also tested in a panel of 29 cell lines (derived from bladder, lung, colon, CNS, melanoma, ovarian, renal, prostate, mammary, gastric, pancreatic, pleural mesothelioma, and uterus tumours), displaying cytotoxicity against all tested cell lines with IC_50_ values in the range 4.2–40.6 µg/mL [63].

#### 2.1.7. Oxadiazole

##### 1,3,4-Oxadiazoles

**Figure 15:**
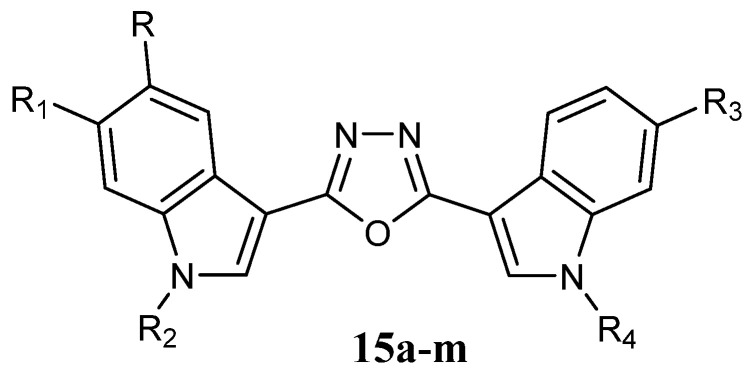
Bis-indolyl-1,3,4-oxadiazole derivatives **15**.

The 1,3,4-oxadiazole scaffold is common to many anticancer agents and ensures cytotoxic properties [65]. Certain 1,3,4-oxadiazole compounds have been identified as tubulin-binding agents and DNA intercalators. Synthesized bis-indolyl-1,3,4-oxadiazoles **15a–m** (Figure 15) were studied for their cytotoxic activity against six human cancer cell lines: pancreas (AsPC1), prostate (DU145 and PC3), cervical (HeLa), breast (MDA-MB-231), and ovarian (OVCAR). Most of the compounds showed strong anticancer activities, with IC_50_ values in the micromolar to nanomolar range (Table 20). The structure-activity relationship study proved that a bromo substituent is decisive for imparting potent cytotoxicity. Bromo-substituted 1,3,4-oxadiazole **15b** was the most active compound in the series, with IC_50_ values of 20 nM against prostate (DU145) and cervical (HeLa) cancer cell lines. In addition, N-alkylation promoted the selectivity of the compound towards a particular tumour type. Preliminary studies in MDA-MB-231 breast cancer cells indicated that the mechanism of action of 1,3,4-oxadiazoles **15a**–**m** was pro-apoptotic [66].

**Figure 16:**
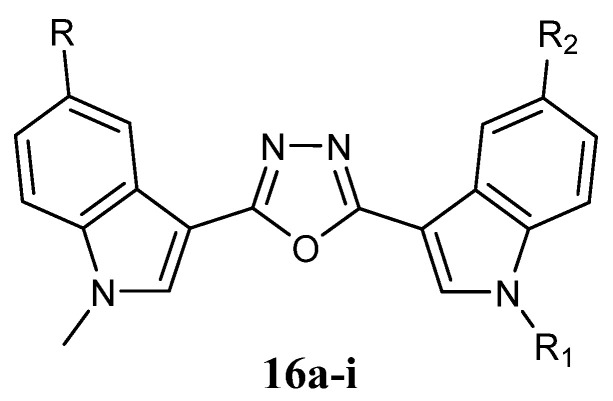
Bis-indolyl-1,3,4-oxadiazole derivatives **16**.

Another 1,3,4-oxadiazole series of type **16** (Figure 16) was synthesised and evaluated for their anti-proliferative activity against lung (A549), breast (MDA-MB-231, MCF-7), and cervical (HeLa) cancer cell lines. The IC_50_ values of the compounds evaluated ranged between 1.8 and 42.3 µM (Table 20). The compounds **16e** and **16h** showed good cytotoxic activity with IC_50_ values of 1.8 µM and 2.6 µM on the breast cancer cell line MCF-7. Three compounds, **16e**, **16f**, and **16h**, showed better cytotoxicity on the cervical cancer cell line (HeLa) with IC_50_ values of 9.23 µM, 9.4 µM, and 6.34 µM. The compound **16h** showed good cytotoxicity with IC_50_ values of 3.3 µM on the lung cancer cell line A549. Moreover, compound **16e** was recognised as a promising drug lead as it showed potent cytotoxicity with an IC_50_ value of 1.8 µM towards MCF-7 when compared to the standard drug doxorubicin (IC_50_ value of 0.98 µM) (Table 21). The compounds **16a**, **16b**, **16g**, and **16i** exhibited moderate cytotoxicity on cervical cancer cell line HeLa. The compound **16d** proved moderate cytotoxicity on breast cancer cell line MCF-7. The compounds **16e** and **16h** displayed moderate cytotoxicity on breast cancer cell line MDA-MB-231 with an IC_50_ value of 12.17 µM and 10.23 µM (Table 21). In addition, no cytotoxicity was found on normal human embryonic kidney cells, HEK-293 [67]. The impact of these compounds on the colchicine-binding site of the tubulin polymer was also evaluated using molecular docking studies. They showed an effective role in the inhibition of mitotic spindle formation thereby altering tubulin polymerization. Moreover, a careful investigation of the binding pattern of ligands provided a few specific elements that are consistent with *in vitro* data. The presence of bromo atom on the indole ring of compound **16e** and the H-bond (2.96 Å) of the methoxy group with Lys 254 might be an explanation of the most potent anti-proliferative activity of **16e** compared to **16a**, **16b**, **16c**, and **16d**. Regarding compound **16h**, the absence of an N-methyl group could be a justifiable reason for the higher potency of compound **16h** compared to compound **16d**. On the other hand, the NH of the free indole ring of compound **16f** was observed to be involved in H-bond with Cy241 (3.15 Å) and Val238 (3.25 Å) which may be the cause of its exhibiting the least activity [67].

##### 1,2,4-Oxadiazoles

**Figure 17:**
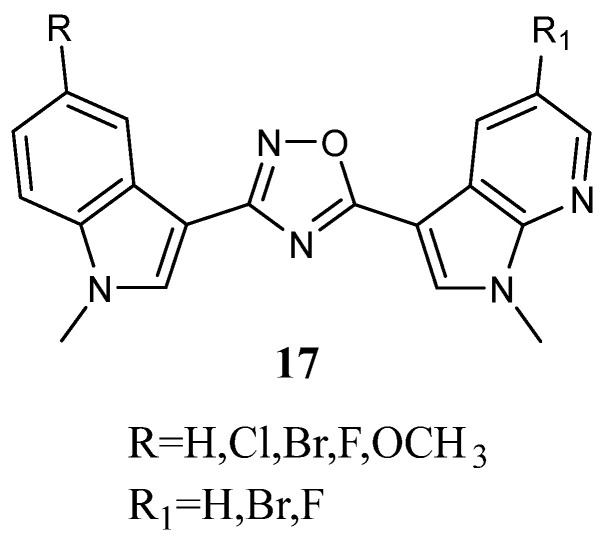
Indolyl-1,2,4-oxadiazol-7-azaindole derivatives **17**.

The 1,2,4-oxadiazole ring system is a five-membered heterocycle ring found in many molecules with significant biological activity, especially anti-tumour. This heterocycle is an amide and ester bioisostere that could improve the bioavailability and physiochemical properties of compounds bearing it. Thus, new indolyl-1,2,4-oxadiazol-7-azaindole Nortopsentin analogs **17** (Figure 17), in which the 1,2,4-oxadiazole ring replaced the imidazole central ring and a 7-azaindole portion substituted the indole, were prescreened against the HCT-116 cell line (colon rectal carcinoma). Two compounds bearing the 5-bromo-1-methyl-7-azaindole moiety (**17a**,**b)** showed the highest cytotoxic activity having IC_50_ < 10 µM (IC_50_ values of 1.93 and 3.55 µM, respectively; Table 22) [68].

Interestingly, the substitution of the bromine atom on the 7-azaindole with fluorine or the absence of the halogen atom resulted in a decrease in the antiproliferative effect. The most active compounds **17a**,**b** were selected for further investigations in additional human tumour cell lines, such as MCF-7 (breast cancer), HeLa (cervical cancer), and CaCo2 (colorectal carcinoma) cell lines, showing IC_50_ values in the micromolar and submicromolar range (Table 22). Compared to **17b**, derivative **17a** appeared more effective [62]. The mechanism of the anti-proliferative effect on MCF-7 was pro-apoptotic, being associated with the externalisation of plasma membrane phosphatidylserine, chromatin condensation, and membrane blebbing. The analysis showed that both compounds led to early apoptosis without causing necrosis. These compounds induced an increase in cells in the G0–G1 phase suggesting that they can act to promote DNA duplication. Moreover, the non-toxicity of these derivatives (**17a**,**b**) was confirmed by experiments on intestinal normal-like differentiated Caco2 cells [68].

#### 2.1.8. Pyrazoles

**Figure 18:**
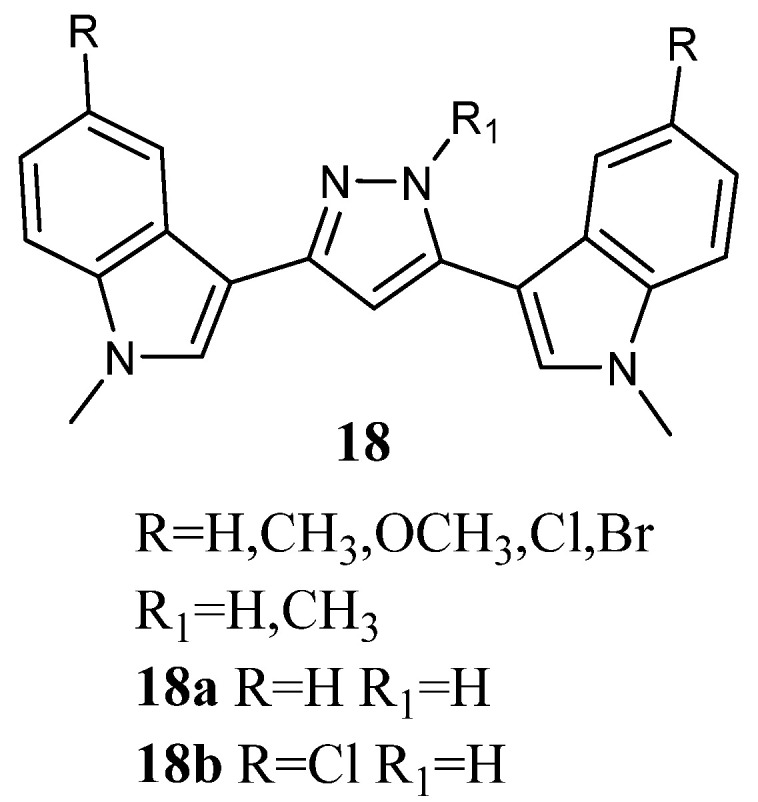
Bis-indolyl-pyrazole derivatives **18**.

Bis-indolyl-pyrazoles **18** (Figure 18), in which a pyrazole central ring substituted the imidazole ring of Nortopsentin, were screened by the NCI.

Among the investigated compounds, derivatives **18a** and **b** were the most active, exhibiting antiproliferative activity against most of the human cell lines. The percentage of sensitive cell lines out of the total number of cell lines investigated was 90% and 100%, respectively, while MG_MID was 18.2 and 3.23 µM, respectively. Therefore, compound **18b**, bearing a chlorine atom, was more active than the unsubstituted derivative **18a**. Derivative **18b** was cytotoxic against the totality of cell lines investigated at micromolar concentration, and it proved to be selective for the melanoma subpanel, having all the subpanel cell lines GI_50_ values in the range of 1.63–9.64 µM (Table 23). The most sensitive cell lines were UACC-62, LOX IMVI, and SK-MEL-5 (GI_50_ values of 1.63, 1.70, and 1.79 µM, respectively). It also showed selectivity for MOLT-4, SR, and K-562 (GI_50_ values of 1.55, 2.36, and 2.78 µM, respectively) of the leukaemia subpanel, HCC-2998 and COLO 205 (GI_50_ values of 1.71 and 2.22 µM, respectively) of colon cancer, CAKI-1 (GI_50_ value of 1.70 µM) of renal cancer, BT-549 (GI_50_ value of 2.03 µM) of breast cancer, and SF-539 (GI_50_ value of 1.81 µM) of CNS subpanel. Derivative **18a** was particularly effective against the colon subpanel, having GI_50_ in the range of 4.58–19.0 µM. The most sensitive colon cell lines were KM12 and HCC-2998 (GI_50_ values of 4.58 and 4.74 µM, respectively). Compound **18a** showed good selectivity for HOP-92 (GI_50_ 2.06 µM) and NCIH460 (GI_50_ value of 4.48 µM) of the non-small-cell lung cancer subpanel and MCF-7 (GI_50_ value of 3.95 µM) of the breast cancer subpanel (Table 23) [60]. Experiments aimed at evaluating the ability of derivatives **18a** and **b** to interact with DNA, revealed that they were unable to form a molecular complex with the macromolecule. In particular, linear flow dichroism spectra obtained by salmon testes DNA, where different concentrations of **18a** and **b** were used, resulted in nearly overlapping to those recorded without compounds. Furthermore, the ability to interfere with the activity of the nuclear enzyme topoisomerase II, which catalyses the interconversion of different topological forms of DNA, was assayed. As to **18a**, the results highlight that the inhibitory capability became detectable at about 50 µM, while in the case of **18b**, it happened at 100 µM. Furthermore, both tested compounds presented a scored inhibition, which was considerably weaker than that of *m*-amsacrine used at 8 µM concentration. These results proved that DNA cannot be considered the main target of cell death, suggesting that other cellular molecular targets are engaged in the antiproliferative activity of **18a** and **b** [63].

#### 2.1.9. Pyrazinones, Pyrazines, Pyrimidines, and Pyridines

**Figure 19:**
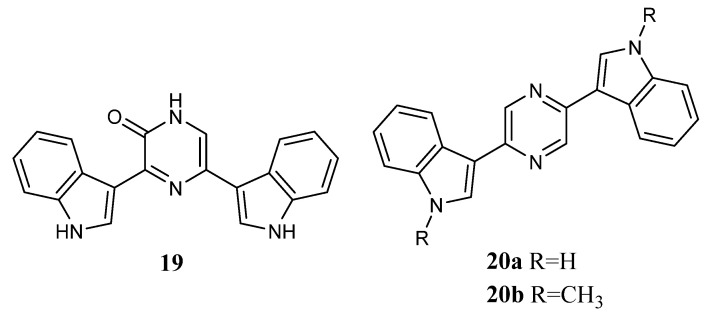
Bis-indolyl-pyrazinone **19** and bis-indolyl-pyrazine derivatives **20a**,**b**.

Bis-indolyl-pyrazinone **19** and bis-indolyl-pyrazines **20a**,**b** (Figure 19) showed inhibitory activity against a variety of human tumour cell lines with GI_50_ values that reached submicromolar levels. In particular, the pyrazinone derivative **19** was active against all tested cell lines (GI_50_ values range of 6.60–74.8 µM), except for the OVCAR-4 cell line; the pyrazine derivative **20a** was active against all cell lines with GI_50_ values range of 2.47–15.5 µM, whereas the N-indolyl methylated compound **20b** was the most active, showing GI_50_ values between 0.058 and 7.19 µM (Table 24) [55,63].

**Figure 20:**
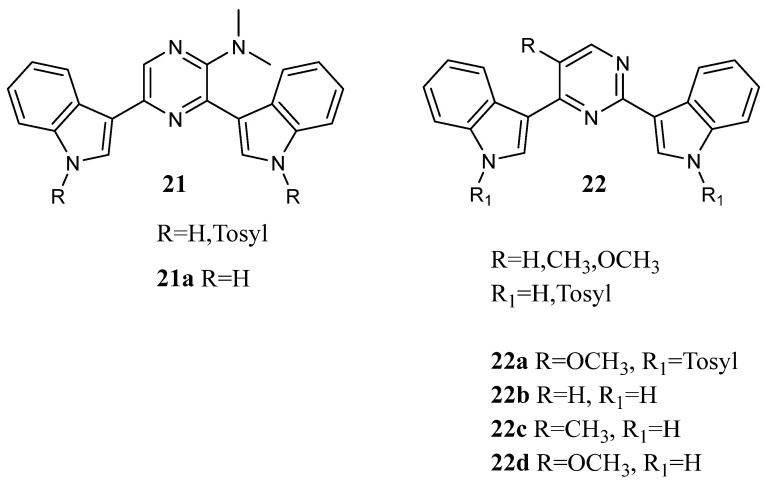
Bis-indolyl-pyrazines **21** and indolyl-pyrimidines **22**.

Novel indolyl-pyrazines **21** and indolyl-pyrimidines **22** (Figure 20) have been synthesized as potential anti-tumour agents. They were *in vitro* screened by NCI in a panel of 60 human tumour cell lines. Compounds **21a**, **22a**–**d** exhibited efficient cytotoxic activities with GI_50_ values in the low micromolar range against a variety of human cancer cell lines. Among these compounds, the pyrimidine **22a** exhibited significant inhibitory activity against leukaemia SR, CNS Cancer SF-539, and breast cancer MDA-MB-435 cell lines with GI_50_ values of 0.22, 0.16, and 0.22 μM, respectively (Table 25). Derivatives **22b** and **c** demonstrated good inhibitory effects against a variety of tumour cell lines with GI_50_ values less than 10 μM. Moreover, the pyrimidine **22b** displayed selective cytotoxicity against IGROV1 tumour cell line with the GI_50_ value below 0.01 μM (Table 25). On the other hand, compound **22d** was active against all tested cell lines with GI_50_ values in the range of 1.13–9.53 μM. Bis-indolyl-pyrazine **21a** also exhibited good inhibitory effects against a variety of tumour cell lines with GI_50_ values less than 10 μM [69].

**Figure 21:**
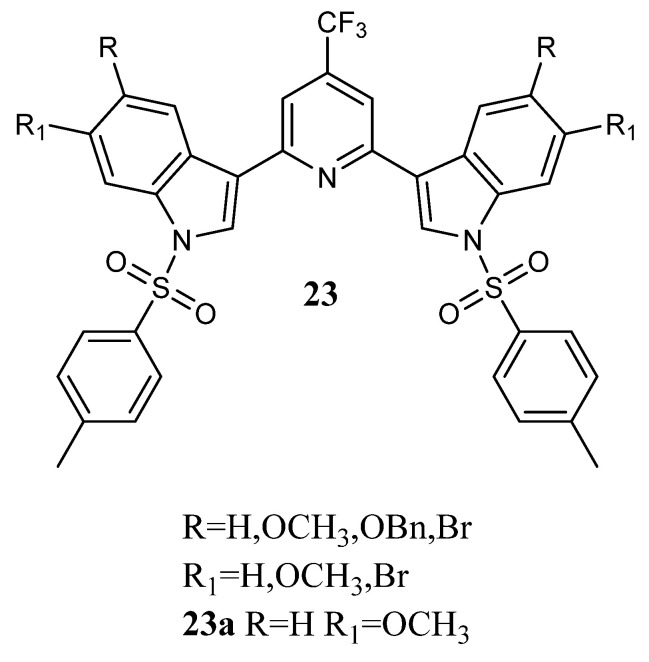
Bis-indolyl-4-trifluoromethyl-pyridines **23**.

In addition, bis-indolyl-4-trifluoromethyl-pyridines **23** (Figure 21) were synthesized and tested against P388 (leukaemia) and A549 (lung cancer) cell lines. Only compound **23a** was active, showing GI_50_ values of 4.3 and 1.7 µM, respectively [55].

### 2.2. Nortopsentins and Bis-Indolyl Compounds as Anti-Inflammatory Agents

**Figure 22:**
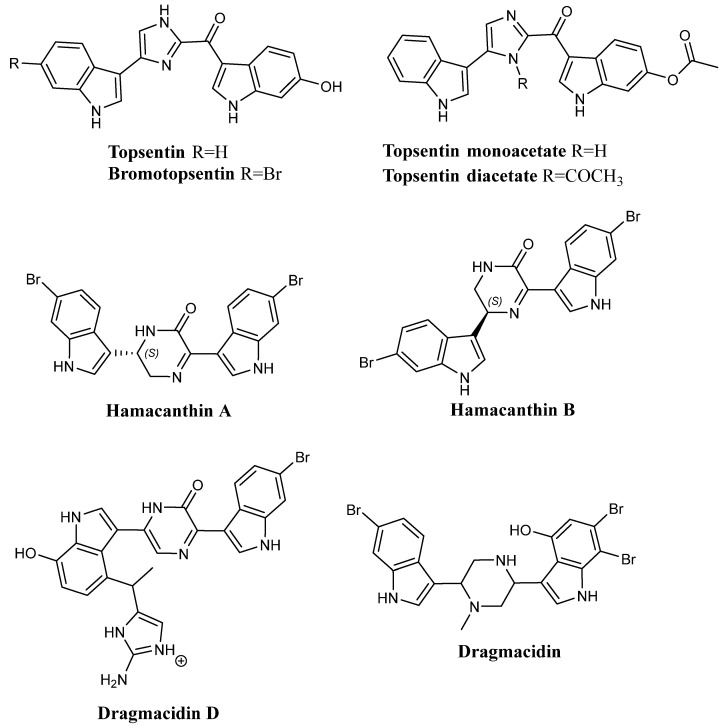
Nortopsentin structurally related to bis-indolyl compounds.

Many natural products, including polysaccharides, phenols, terpenoids, quinones, and alkaloids, have proven to be potential anti-inflammatory agents, among which emerge marine alkaloids derived from different marine sources, such as sponges, bryozoans, and fungus [70,71,72,73,74,75,76]. Nortopsentin A (Figure 1), Nortopsentin B (Figure 1), and Nortopsentin C (Figure 1), together with the structurally related bis-indolyl compounds Topsentin (Figure 22), Bromotopsentin (Figure 22), Topsentin monoacetate (Figure 22), Topsentin diacetate (Figure 22), Dragmacidin (Figure 22), Hamacanthin A (Figure 22), Hamacanthin B (Figure 22) have been found to have significant anti-inflammatory properties. These compounds have been screened by standard anti-inflammatory assays. In particular, when tested by mouse ear anti-inflammatory assay using phorbol myristate acetate (PMA), Topsentin showed greater potency (ED_50_ = 15 µg/ear) than the known anti-inflammatories hydrocortisone, Indomethacin, and Manoalide (ED_50_ of 20, 250, and 100 µg/ear, respectively). In addition, bis-indolyl compounds were tested in order to calculate the percentage of PMA-induced oedema inhibition. Data revealed that Topsentin, Bromotopsentin, Dragmacidin, Nortopsentin A, and Nortopsentin C displayed significant potency (Table 26), with inhibition percentages reaching 98.1 and 70.1% in the case of Nortopsentin A and C [77].

Topsentin also proved to be capable of inactivating bee venom phospholipase A2 with IC_50_ lower than hydrocortisone and Indomethacin (0.5 µM, >1 mM, and >1 mM, respectively). Bis-indolyl compounds were tested at a final concentration of 1 μΜ to determine the percentage of bee venom phospholipase A2 inactivation. The results of this test are shown in Table 27; the phospholipase A2 inactivation percentage reached 67% in the case of Topsentin.

In consideration of the data presented, the aforementioned bis-indolyl compounds showed potent anti-inflammatory effects. Their mechanism of action appears to be the consequence of phospholipase A2 inactivation. Moreover, for Topsentin, the percentage of bee venom phospholipase A2 inactivation was found equal to 80% when tested at a final concentration of approximately 2 μΜ. In the mouse ear oedema inhibition assay, a dose of about 12 µg/ear of Topsentin achieved nearly 50% oedema inhibition, and doses of 100 µg/ear showed more than 90% oedema inhibition. Similarly, oedema inhibition percentages induced by Bromotopsentin ranged from about 20% for a dose of approximately 25 µg/ear to about 75% at a dose of 50 µg/ear [77,78].

Other inflammatory assays, such as mouse ear anti-inflammatory assay using resiniferatoxin (RTX), a neurogenic inflammation-producing compound, provided the ability of Nortopsentin C (Figure 1), Hamacanthin B (Figure 22), and Topsentin (Figure 22) to inhibit RTX-induced oedema. At a concentration of 50 µg/ear, oedema inhibition percentages of 98.4%, 96.9%, and 82%, respectively, for Nortopsentin C, Hamacanthin B, and Topsentin (Table 28), with ED_50_ values of 8 µg/ear, 1.5 µg/ear, and 20 µg/ear (Table 29) [48]. These data are consistent with the experiments previously discussed, supporting the claim that Nortopsentin and bis-indolyl analogues have potent anti-inflammatory activity through oedema inhibition.

Moreover, Nortopsentin C (Figure 1) and the related compounds Dragmacidin (Figure 22), Hamacanthin A (Figure 22), Dragmacidin D (Figure 22), and Topsentin (Figure 22) were discovered to be neural nitric oxide synthase (bNOS) inhibitors. They inhibited rat bNOS activity with IC_50_ values of 27 µM, 20 µM, 7.5 µM, and 4 µM, respectively, while Tosentin showed minimal inhibitory effects. The bNOS inhibition percentage induced by these compounds at a concentration range of 1.0–50 µM was also measured. The most potent inhibitory activity was shown by Hamacanthin A, Dragmacidin D, Nortopsentin C, and Dragmacidin at the concentration of 50 µM with inhibition percentages of 99.58%, 99.56%, 98.77%, and 90.27%, respectively (Table 30). Furthermore, among these compounds, Nortopsentin C and Dragmacidin were the only ones that exhibited potent calcineurin inhibition with IC_50_ values of 11.4 µM and 10 µM [51]. This suggests that the likely target of Nortopsentin C and Dragmacidin is calmodulin, a co-factor common to bNOS and calcineurin. These experiments prove the usefulness of bis-indolyl compounds, including Nortopsentin C, in therapeutic applications for the treatment of neurodegenerative disorders and inflammatory reactions.

**Figure 23:**
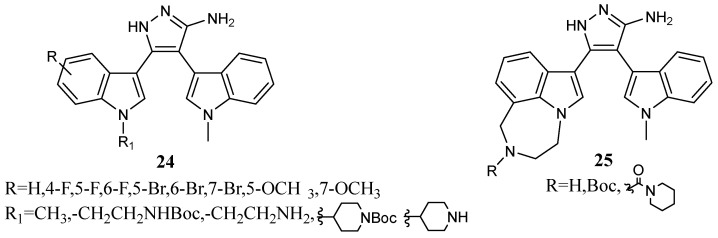
Aminopyrazole derivatives **24** and **25** with the Nortopsentin-like scaffold.

Recent research also disclosed that some GSK3β inhibitors with the Nortopsentin-like scaffold (**24a**, **24b**, **24c**, **25a**, and **25b**, Figure 23, Table 31), exhibit inhibitory effects on microglial inflammation and oxidative neurotoxicity. After exposure to 100 ng/mL LPS for 24 h and preincubation with the compounds **24a**, **24b**, **24c**, **25a**, and **25b**, a significant suppression of the LPS-induced NO production was observed in BV-2 cells (a well-known microglial inflammatory cellular model). Among these tested compounds, **24c** and **25a** at 20 μM exhibited the most potent anti-inflammatory properties, by showing an approximately 50% reduction in NO release, as compared to the LPS alone group. In addition, in order to evaluate the protective effects of compounds **24a**, **24b**, **24c**, **25a**, and **25b** against glutamate-induced oxidative neuronal damage, HT-22 cells were treated with these compounds for 2 h before glutamate exposure. This experiment revealed that pretreatment with 1 and 10 μM of compounds **24a**, **24b**, and **24c** induced potent protective effects with cell viability percentages greater than 80%. Compounds **25a** and **25b** at 10 μM also markedly relieved the oxidative neuronal damage in HT-22 cells, while no noticeable protection was noticed at a lower concentration (1 μM) [79].

The most promising compound, **24c**, was selected for further *in vivo* study. Microglial activation and astrocyte proliferation in the brain of LPS-injected mice were measured by IBA-1 and GFAP immunofluorescence staining. Results indicated that compound **24c** markedly reduced microglial activation and astrocyte proliferation, showing a potent anti-inflammatory effect. This new potent GSK3β inhibitor could be the starting point for the discovery of therapeutic agents to treat Alzheimer’s disease and other inflammation-associated neurological syndromes [79].

## 3. Conclusions

Synthesizing marine compound analogues appears to be a promising strategy to obtain new anticancer derivatives that act by intervening in specific cell processes. On the one hand, most studies on Nortopsentin derivatives have focused on the antiproliferative activity evaluation and action mechanisms commonly involved in cancer. Nortopsentin derivatives can induce cell cycle arrest and apoptosis in cancer cell lines and simultaneously impair cell viability. The driving element of their antiproliferative activity is mostly overexpressed target inhibition, such as the kinases CDK1 and GSK3β or enzymes like GLS-1. The most active compounds, showing GI_50_ values in the micromolar-submicromolar range in different human tumour cell lines, belong to the thiazole class, in which the structural manipulation of Nortopsentin was extended to one of the two indolyl portions that were replaced by a 7-azaindole ring. The five most active compounds, which were further investigated in two additional cell lines, STO and MesoII, derived from human diffuse malignant peritoneal mesothelioma (DMPM), exhibited IC_50_ values ranging from 0.33 to 0.61 μM in STO cells and from 4.11 to 25.12 μM in MesoII. Moreover, their anti-tumour activity was evaluated on STO cells xenotransplanted in athymic nude mice. The treatment with the different compounds resulted in marked tumour growth inhibition with two complete responses (disappearance of tumour) in each treatment group without any appreciable sign of toxicity. *In vitro* kinase assays revealed CDK1 inhibition exerted by the compounds with IC_50_ values lower than 1 μM. On the other hand, Nortopsentin and structurally related compounds also exhibit anti-inflammatory properties, such as anti-oedema and neuroprotection. *In vivo* anti-inflammatory assays on animal models disclose that Nortopsentins can powerfully inhibit oedema; in particular, Nortopsentin A, and Nortopsentin C, when tested by mouse ear anti-inflammatory assay using phorbol myristate acetate (PMA), displayed significant potency with inhibition percentages reaching 98.1 and 70.1%, respectively. The key factor behind this effect is phospholipase A2 inactivation. In addition, Nortopsentin C mightily inhibits rat neural nitric oxide synthase (bNOS) and calcineurin with IC_50_ values of 27 and 11.4 µM, respectively. Some Nortopsentin analogue GSK3β inhibitors, bearing an aminopyrazole central ring instead of the imidazole of the lead, also suppress LPS-induced NO production in mice, providing powerful anti-inflammatory effects. Overall, Nortopsentins emerge as new lead compounds for the development of novel anti-inflammatory agents, indicating the need to synthesise new Nortopsentin derivatives and investigate their antiproliferative activity as well as their anti-inflammatory potential.

## Figures and Tables

**Table 1:** Marketed drugs by FDA.

Compound	Date of FDA Authorisation	Natural Source	Clinical Use
Cytarabine	1969	Sponge	Leukaemia
Fludarabine	2004	Sponge	Leukaemia
Trabectedin	2015	Tunicate	Ovarian cancer
Eribulin	2010	Sponge	Breast cancer
Brentuximab vedotin	2011	Mollusk/cyanobacterium	Lymphomas
Lurbinectedin	2020	Tunicate	Ovarian cancer
Polatuzumab vedotin	2019	Mollusk/cyanobacterium	Breast cancer
Enfortumab vedotin	2019	Mollusk/cyanobacterium	Urothelial cancer
Belantamab mafodotin	2020	Mollusk/cyanobacterium	Multiple myeloma

**Table 2:** *In vitro* inhibition of cancer cell lines growth in leukaemia, colon, CNS, ovarian, and breast cancer subpanels by compounds **1**.

Cell Line	GI_50_ (µM) ^a^									
	1a	1b	1c	1d	1e	1f	1g	1h	1i	1j
Leukaemia										
CCRF-CEM	ND ^b^	14.6	10.9	10.1	2.11	2.40	27.7	2.58	2.66	2.99
HL-60 (TB)	ND ^b^	ND ^b^	ND ^b^	0.95	2.43	2.76	ND ^b^	3.76	4.13	3.86
K-562	3.27	18.8	5.61	4.69	1.96	1.94	15.2	2.13	1.74	2.15
MOLT-4	5.31	19.9	31.2	5.80	1.41	1.75	23.0	1.55	2.95	2.26
RPMI82226	ND ^b^	19.4	12.2	11.4	1.97	1.95	27.1	2.24	2.03	1.84
Colon Cancer										
HCT-15	>100	15.2	17.8	8.5	1.81	2.51	7.54	2.70	0.81	2.84
SW-620	>100	16.5	25.6	12.5	1.52	2.14	7.00	3.57	5.50	2.89
CNS Cancer										
SF-295	33.6	14.6	9.23	4.81	1.98	2.71	73.5	4.56	ND ^b^	ND ^b^
SF-268	ND ^b^	18.3	32.1	ND ^b^	1.52	2.44	26.0	2.69	13.8	1.80
SNB-19	>100	17.8	41.9	17.2	2.11	3.75	>100	2.60	10.5	3.34
U251	>100	17.9	28.1	15.3	2.10	2.30	25.0	3.34	5.07	3.00
Ovarian Cancer										
IGROV1	8.14	13.0	30.5	14.4	1.85	1.70	81.5	2.96	4.61	2.43
OVCAR-5	>100	16.1	37.1	23.4	1.96	2.14	42.7	2.16	3.44	2.35
Breast Cancer										
MCF-7	>100	16.7	27.2	6.5	2.13	2.70	54.1	0.88	4.36	3.82
MDA-MB-435	33.1	14.9	25.6	4.3	2.53	2.27	7.70	4.54	14.6	3.97
MDA-N	83.0	19.2	31.6	2.9	1.88	2.09	8.27	2.86	6.84	3.77
T-47D	>100	24.8	23.9	16.2	3.27	2.90	59.3	3.33	4.12	1.76
BT-549	>100	18.3	73.8	41.1	2.71	10.6	67.3	1.24	1.46	1.41

^a^ Concentration (µM) that inhibits 50% net cell growth. ^b^ ND = Not Determined.

**Table 3:** Cytotoxic activity of compounds **2c**, **2d**, **2e**, **2g**, and **2m** in DMPM and normal cells.

Compound	IC_50_ (µM) ^a^		
	STO	MesoII	W138
**2c**	0.49 ± 0.07	25.12 ± 3.06	>100
**2d**	0.61 ± 0.14	16.77 ± 1.99	18.76 ± 3.21
**2e**	0.43 ± 0.11	4.85 ± 0.64	>100
**2g**	0.54 ± 0.09	13.27 ± 0.74	15.44 ± 3.87
**2m**	0.33 ± 0.07	4.11 ± 0.22	>100

^a^ Data are reported as IC_50_ values (concentration of drug required to inhibit growth by 50%) determined by MTS assay after 72 h of continuous exposure to each compound. The data represent mean values ± SD of at least three independent experiments.

**Table 4:** Activity of derivatives **2c**, **2e**, and **2m** on STO cells xenotransplanted in athymic nude mice.

Compound	TVI (%) ^a^	CR ^b^	BWL (%) ^c^	TOX ^d^
**2c**	73 *	2/8	4	0/8
**2e**	75 **	2/8	1	0/8
**2m**	58 *	2/8	7	0/8

^a^ Tumour volume inhibition (%) in treated vs. control mice, determined 17 days after the end of drug treatment (day 35). ^b^ Complete response, disappearance of tumour induced by treatment. ^c^ BWL, body weight loss induced by treatment (%). ^d^ Toxic death on treated animals. ** *p* < 0.01, * *p* < 0.05.

**Table 5:** *In Vitro* kinase inhibitory properties of derivatives **2c**, **2e**, and **2m**.

Protein Kinase		IC_50_ (µM) ^a^	
	2c	2e	2m
CDK1	0.89 ± 0.07	0.75 ± 0.03	0.86 ± 0.04
GSK3β	42.18 ± 3.28	40.18 ± 2.94	35.68 ± 1.69

^a^ Concentration of drug required to inhibit by 50% (IC_50_) the activity of CDK1 and GSK3β. The data represent mean values ± SD of at least three independent experiments.

**Table 6:** Cytotoxic activity and the selectivity index (SI) of the synthesized compounds **2n**–**q** against MCF-7 cancer cell line.

Compound	Intestinal Normal-like Caco-2 Cells LC_50_ (µM) ^a^	MCF-7 GI_50_ (µM) ^b^	SI
**2n**	>100	0.05	ND ^c^
**2o**	96.1 ± 3.1	2.09	46
**2p**	71.4 ± 2.1	1.74	41
**2q**	83.3 ± 2.5	1.79	46

^a^ LC_50_ concentration which is lethal to 50% of the normal cells compared to untreated controls; ^b^ GI_50_ Concentration (µM) that inhibits 50% net cell growth; ^c^ ND, not determined.

**Table 7:** *In vitro* inhibition of cancer cell lines growth in leukaemia, colon, renal, and breast cancer subpanels by compounds **2t**–**w**, **2z**, **2ab**, **2ae**, and **2ak**–**am**.

Cell Line	GI_50_ (µM) ^a^									
	2t	2u	2v	2w	2z	2ab	2ae	2ak	2al	2am
Leukaemia										
CCRF-CEM	2.76	1.94	2.12	2.25	2.12	1.83	3.07	0.45	1.97	1.73
HL-60(TB)	2.11	1.88	1.77	1.86	1.93	1.72	2.52	1.67	1.80	1.70
K-562	2.08	1.74	1.72	1.88	1.95	1.61	2.94	0.24	1.49	1.49
MOLT-4	1.85	1.82	1.83	1.80	1.86	1.77	1.79	0.60	1.79	1.54
RPMI82226	1.92	2.08	2.04	2.19	1.99	1.84	1.68	0.77	1.84	1.89
SR	2.30	1.89	2.05	1.84	2.01	1.68	2.29	0.27	1.70	1.58
Colon Cancer										
COLO-205	1.82	1.67	1.73	1.80	1.85	1.73	2.09	1.49	1.78	1.63
HCC-2998	2.04	1.71	1.70	1.78	1.91	1.74	3.42	1.47	1.59	1.86
HCT-116	1.85	1.70	1.75	1.68	1.78	1.60	3.00	0.28	1.83	1.37
HCT-15	2.32	1.72	1.73	1.57	1.96	1.58	2.28	0.24	1.70	1.45
HT29	2.22	1.75	1.68	1.81	2.18	1.56	2.85	0.31	1.70	1.59
KM12	2.41	1.61	1.77	1.87	1.93	1.73	2.85	1.09	1.69	1.70
SW-620	2.26	1.82	1.92	2.00	1.99	1.74	3.62	0.31	1.83	1.62
Renal Cancer										
786-0	1.74	1.63	1.65	1.61	1.78	1.59	2.91	0.42	1.65	1.61
A498	0.48	1.83	2.57	1.82	0.22	1.43	5.66	0.02	1.60	1.66
ACHN	2.50	1.66	1.70	1.62	1.79	1.66	2.82	1.50	1.73	165
CAKI-1	1.88	ND ^b^	ND ^b^	ND ^b^	1.80	ND ^b^	ND ^b^	1.39	ND ^b^	ND ^b^
RXF 393	2.09	1.62	1.61	1.59	1.73	1.38	2.58	0.93	1.52	1.33
SN12C	3.16	1.68	1.74	1.75	1.91	1.60	3.15	6.05	1.53	1.61
TK-10	3.48	2.22	1.85	1.59	5.25	1.51	3.28	4.25	1.58	1.53
UO-31	1.37	1.18	1.28	1.38	1.43	1.38	2.20	1.11	1.46	1.43
Breast Cancer										
MCF-7	1.43	1.24	1.53	1.43	1.56	1.53	1.94	1.65	1.62	1.70
MDA-MB-231/ATCC	1.86	1.63	1.69	1.66	1.70	1.61	2.51	0.52	1.64	1.64
HS 578T	2.08	2.34	2.59	2.05	2.05	1.66	3.75	0.76	12.40	1.50
BT-549	2.00	1.68	3.72	3.28	3.74	1.66	2.17	2.97	1.67	1.64
T-47D	2.07	1.22	1.77	1.75	2.12	1.45	1.52	12.0	15.70	1.65
MDA-MB-468	2.52	1.88	1.61	1.86	1.95	1.68	2.20	0.97	1.51	1.50

^a^ Concentration (µM) that inhibits 50% net cell growth. ^b^ ND = Not Determined.

**Table 8:** Toxicity of the compound **2y** on aggressive cancer cell lines with GLS-1 overexpression.

Cell Line	EC_50_ ± SD (µM) ^a^
U-87 MG	3.63 ± 1.87
MIA PaCa-2	6.98 ± 0.44
Saos2	4.85 ± 1.19
A-375	5.84 ± 0.43
A549	8.47 ± 0.32

^a^ EC_50_ values are expressed as mean ± SD.

**Table 9:** *In vitro* inhibition of cancer cell lines growth by compounds **3a**–**h**.

Cell Line	GI_50_ (µM) ^a^							
	3a	3b	3c	3d	3e	3f	3g	3h
Leukaemia								
CCRF-CEM	2.02	1.78	1.83	2.15	1.30	0.36	1.74	1.96
HL-60(TB)	2.03	1.94	1.68	1.96	1.71	1.48	1.76	1.93
K-562	1.76	2.09	0.72	1.90	0.24	0.24	0.46	1.70
RPMI82226	2.00	1.76	1.83	1.84	1.58	1.57	1.77	1.98
Colon Cancer								
HCC-2998	1.22	1.26	1.85	1.89	1.76	1.90	1.64	1.86
HCT-116	1.61	0.93	1.69	1.90	1.21	0.18	1.63	1.66
HCT-15	1.37	1.51	1.25	1.54	1.35	1.75	1.33	1.68
HT29	1.82	1.98	1.20	1.43	1.59	1.60	1.44	1.47
KM12	1.11	1.00	1.73	1.84	1.71	1.97	1.63	1.79
SW-620	2.03	1.85	1.75	1.96	1.52	1.85	1.65	1.88
Breast Cancer								
MCF-7	0.30	0.32	1.47	1.70	1.33	1.78	1.23	1.93
MDA-MB-231/ATCC	1.56	1.47	1.45	1.83	1.41	1.66	1.55	1.75
HS 578T	1.93	2.16	2.03	2.21	1.94	2.23	2.21	2.36
BT-549	9.00	1.68	1.81	1.80	1.93	1.61	1.80	1.71
MDA-MB-468	0.40	0.27	0.44	1.62	1.38	1.79	1.40	1.86

^a^ Concentration (µM) that inhibits 50% net cell growth.

**Table 10:** *In vitro* inhibition of cancer cell lines growth by compounds **3i**–**p**.

Cell Line	GI_50_ (µM) ^a^							
	3i	3j	3k	3l	3m	3n	3o	3p
Leukaemia								
CCRF-CEM	1.89	1.75	1.44	2.40	2.45	2.33	2.24	0.42
HL-60(TB)	2.07	2.19	2.02	2.17	1.51	1.90	2.09	2.11
K-562	0.37	1.48	2.05	1.85	1.72	1.78	0.98	0.35
RPMI82226	1.77	1.86	1.25	2.14	2.08	2.02	2.48	2.13
Colon Cancer								
HCC-2998	1.92	1.95	1.87	2.01	1.87	1.88	1.98	2.10
HCT-116	1.61	1.22	1.71	1.73	1.77	1.77	1.66	1.56
HCT-15	1.33	1.67	1.56	1.79	1.95	1.88	1.65	1.38
HT29	1.35	1.69	2.07	2.01	2.23	1.53	1.71	1.57
KM12	1.67	1.83	2.02	1.65	1.76	1.87	1.91	2.33
SW-620	1.88	2.12	1.73	1.98	1.93	1.95	2.04	1.81
Breast Cancer								
MCF-7	1.48	1.27	1.43	1.22	1.66	1.81	1.53	1.77
MDA-MB-231/ATCC	1.27	1.46	1.06	1.67	1.65	1.87	1.52	2.34
HS 578T	1.71	2.26	2.00	2.21	2.15	14.7	2.23	2.47
BT-549	1.65	1.63	8.82	1.78	8.96	1.79	1.86	26.3
MDA-MB-468	1.51	1.45	0.23	ND ^b^	1.78	1.97	1.77	1.68

^a^ Concentration (µM) that inhibits 50% net cell growth. ^b^ ND = Not Determined.

**Table 11:** Overview of the results of the *in vitro* anti-tumour screening for compounds **4a**, **4b**, **5a**, **6a**, and **7a**–**7g**.

Thiazole Derivative	N° Cell Lines Tested	N° Active Cell Lines	GI_50_ Range (µM) ^a^
**4a**	60	60	0.03–13.0
**4b**	59	59	0.04–14.2
**5a**	54	48	0.81–27.7
**6a**	59	59	0.93–4.70
**7a**	58	29	4.11–6.89
**7b**	59	59	4.45–5.98
**7c**	59	59	4.63–5.28
**7d**	60	60	4.87–7.50
**7e**	60	42	4.03–6.11
**7f**	59	9	4.01–7.56
**7g**	59	59	4.64–5.59

^a^ Concentration range that inhibits 50% net cell growth.

**Table 12:** Cytotoxic activity of compounds **7b**–**d**,**7g** in MiaPaCA-2 and STO cell lines.

Compound	IC_50_ (µM) ^a^	
	MiaPaCA-2	STO
**7b**	41.6 ± 2.4	17.2 ± 2.9
**7c**	5.7 ± 0.8	0.83 ± 0.04
**7d**	4.3 ± 0.6	0.41 ± 0.06
**7g**	39.6 ± 3.9	5.7 ± 0.8

^a^ Concentration of drug required to inhibit growth by 50% as determined by SRB assay after 72 h continuous exposure to each compound; the data represent mean values ± SD of at least three independent experiments.

**Table 13:** Kinase inhibition by compounds **7b**–**d**,**6g**.

Compound	IC_50_ (µM) ^a^		
	CDK1	CDK5	GSK3β
**7b**	33.27 ± 2.97	>50.0	>50.0
**7c**	0.41 ± 0.08	>50.0	>50.0
**7d**	0.85 ± 0.13	>50.0	>50.0
**7g**	45.93 ± 4.19	>50.0	>50.0
**Purvanalol A**	0.73 ± 0.06	>50.0	>50.0
**Roscovitine**	0.59 ± 0.08	>50.0	>50.0

^a^ Inhibitor concentration at which enzyme activity is decreased by 50%; data represent the mean ± SD of at least three independent experiments.

**Table 14:** GI_50_ values of the most active compounds **8a**, **9a**, and **9b** on the MCF-7 cells.

Thiazole Derivative	GI_50_ (μM) ^a^
**8a**	2.13 ± 0.12
**9a**	3.26 ± 0.19
**9b**	5.14 ± 0.34

^a^ Concentration (µM) that inhibits 50% net cell growth; data represent the mean ± SD of at least three independent experiments.

**Table 15:** Anticancer activity of the compound **10a** against selected human cancer cell lines IC_50_ (μM)^a^.

Compound	MDA-MB-231	MCF-7	LnCaP	DU145	PC3	PaCa2
**10a**	67.9	32.1	14.6	369.8	21.4	21.2

^a^ IC_50_ values were obtained using a dose–response curve by non-linear regression using a curve fitting program.

**Table 16:** *In vitro* activity of derivatives **12a**–**c** towards 10 human tumour cell lines.

Compound	IC_50_ (µg/mL)	Active/Total ^a^			Tumour Selectivity ^b^
		1 (µg/mL)	10 (µg/mL)	100 (µg/mL)	
**12a**	27.1	0%	0%	70%	0/10
**12b**	21.1	0%	10%	80%	1/10
**12c**	17.1	0%	20%	70%	2/10

^a^ Responsive (T/C < 30%)/total cell lines. ^b^ Selective (individual IC_70_ < 1/3 mean IC70)/total cell lines.

**Table 17:** *In vitro* inhibition of cancer cell lines growth in leukaemia subpanel by compounds **11a** and **12c**.

Cell Line	GI_50_ (µM) ^a^	
	11a	12c
Leukaemia		
CCRF-CEM	0.34	6.46
HL-60(TB)	2.27	1.98
K-562	3.54	2.86
MOLT-4	1.91	>4.00
RPMI82226	2.83	2.25
SR	ND ^b^	1.63

^a^ Concentration (µM) that inhibits 50% net cell growth. ^b^ ND = Not Determined.

**Table 18:** *In vitro* and *ex vivo* anti-tumour activity by derivatives **13a** and **b**: In *in vitro* tumour cell lines (monolayer assay) and in *ex vivo* human xenografts (clonogenic assay).

Cell Line	IC_50_^a^ (μM)		Tumour Histotype	IC_50_^a^ (μM)	
	13a	13b		13a	13b
Bladder					
BXF 1218L	0.72	0.32	BXF 1218	2.45	2.35
BXF 1352L	0.68	0.41	BXF 1228	2.89	2.86
BXF T24	1.72	0.58			
Lung Cancer					
LXFA 289L	6.34	2.39	LXFA 1012	4.76	27.34
LXFA 526L	1.57	0.67	LXFA 1584	2.07	2.73
LXFA 629L	2.28	1.36	LXFA 297	54.90	>100
LXFL 1121L	0.67	0.38	LXFA 526	2.96	3.15
LXFL 529L	1.85	0.67	LXFA 629	2.23	3.63
LXFL H460	2.08	0.89	LXFA 677	23.19	27.02
			LXFA 923	19.50	25.68
			LXFE 1422	5.90	1.72
			LXFL 1072	3.44	3.42
			LXFL 529	5.07	4.46
			LXFL 625	24.04	38.21
Colon Cancer					
CXF 269L	1.39	0.56	CXF 1103	4.47	18.36
CXF HCT116	3.24	1.64	CXF 1729	3.49	7.77
CXF HT29	5.20	2.65	CXF 1783	37.57	40.70
CXF RKO	1.50	0.63	CXF 280	26.18	35.70
			CXF 975	6.93	6.03
Head and Neck					
HNXF CAL27	0.81	0.50	HNXF 536	2.45	2.67
			HNXF 908	2.57	4.00
Melanoma					
MEXF 1341L	0.52	0.19	MEXF 1539	19.90	15.31
MEXF 276L	0.22	0.11	MEXF 276	1.44	1.75
MEXF 462NL	1.31	0.55	MEXF 462	3.24	4.10
			MEXF 989	1.18	0.58
Ovarian Cancer					
OVXF OVCAR3	1.46	0.58	OVXF 1353	20.96	22.90
OVXF 899L	5.40	2.03	OVXF 899	3.54	3.93
Renal Cancer					
RXF 1183L	1.13	0.58	RXF 1220	6.34	3.16
RXF 1781L	1.77	0.66	RXF 486	2.90	3.90
RXF 393NL	3.14	1.34	RXF 631	2.98	2.81
RXF 486L	3.86	1.60			
Prostate Cancer					
PRXF 22RV1	1.46	0.63	PRXF DU145	28.67	25.98
PRXF DU145	4.45	1.96	PRXF PC3M	2.89	2.80
PRXF LNCAP	2.10	0.68			
PRXF PC3M	0.85	0.32			
Mammary Cancer					
MAXF 401NL	1.24	0.64	MAXF 1322	16.80	9.49
MAXF MCF-7	2.44	0.98	MAXF 1384	34.48	33.28
MAXF MDA-231	1.18	0.49	MAXF 401	5.90	11.32
Gastric Cancer					
GXA MKN45	0.84	0.53	GXF 1172	6.27	>100
GXF 251L	1.56	0.65	GXF 251	7.10	25.50
			GXF 97	2.72	3.74
Pancreatic Cancer					
PANC1	0.74	0.41	PAXF 546	3.52	14.63
1657L	2.68	1.02	PAXF 736	2.99	2.10
546L	2.70	1.16			
Pleural mesothelioma					
PXF 1118L	2.50	0.88	PXF 1752L	3.71	4.05
PXF 1752L	0.89	0.43	PXF 541	2.20	0.37
PXF 698L	1.86	0.86			
Sarcoma					
SXF SAOS2	0.72	0.33	SXF 1186	5.71	6.17
SXF TE671	1.60	0.53	SXF 1301	3.54	23.95
			SXF 627	3.40	4.06
Uterus Cancer					
UXF 1138L	0.72	0.35			

^a^ Concentration (μM) that inhibits 50% net cell growth.

**Table 19:** *In vitro* activity of derivatives **14a**–**e** towards 10 human tumour cell lines.

Compound	IC_50_ (µg/mL)	Active/Total ^a^			Tumour Selectivity ^b^
		1 (µg/mL)	10 (µg/mL)	100 (µg/mL)	
**14a**	9.6	0/10 (0%)	2/10 (20%)	10/10 (100%)	1/10
**14b**	44.5	0/10 (0%)	0/10 (0%)	7/10 (70%)	0/10
**14c**	17.3	0/10 (0%)	1/10 (10%)	7/10 (70%)	2/10
**14d**	24.5	0/10 (0%)	0/10 (0%)	7/10 (67%)	1/10
**14e**	43.2	0/10 (0%)	0/10 (0%)	3/10 (30%)	1/10

^a^ Responsive (T/C < 30%)/total cell lines. ^b^ Selective (individual IC_70_ < 1/3 mean IC_70_)/total cell lines.

**Table 20:** Cytotoxicity of oxadiazoles **15a**–**m** against a panel of human cancer cell lines: pancreas (AsPC1), prostate (DU145 and PC3), cervical (HeLa), breast (MDA-MB-231), and ovarian (OVCAR).

Compounds	R	R_1_	R_2_	R_3_	R_4_	IC_50_ (µM) ^a^					
						AsPc1	DU145	PC3	MDA-MB-231	OVCAR	HeLa
**15a**	H	H	H	H	H	0.46	0.23	8.43	0.09	0.10	0.53
**15b**	Br	H	H	H	H	0.20	0.02	0.25	0.24	0.15	0.02
**15c**	OCH_3_	H	H	H	H	0.06	0.72	0.11	0.90	0.38	66.79
**15d**	H	F	H	H	H	0.31	9.79	0.29	4.34	0.31	6.86
**15e**	Br	H	H	F	H	0.45	0.08	4.55	0.20	0.23	0.06
**15f**	OCH_3_	H	H	F	H	1.68	0.42	0.10	6.44	2.91	52.90
**15g**	H	F	H	F	H	0.20	0.15	1.09	0.21	0.15	0.06
**15h**	H	H	CH_3_	H	CH_3_	0.10	0.52	0.12	15.71	0.97	4.55
**15i**	H	H	4-ClC_6_H_4_CH_2_	H	H	2.47	0.37	7.11	126.3	0.16	0.73
**15j**	H	H	4-CH_3_OC_6_H_4_CH_2_	H	H	0.05	0.54	3.33	168.9	0.10	5.94
**15k**	Br	H	4-ClC_6_H_4_CH_2_	H	H	0.43	0.11	0.24	0.86	0.27	8.10
**15l**	H	F	4-ClC_6_H_4_CH_2_	H	H	1.60	0.14	0.05	2.58	0.24	0.96
**15m**	H	H	4-CH_3_OC_6_H_4_CH_2_	H	H	0.07	0.10	0.14	17.22	0.11	1.20

^a^ IC_50_ values were obtained using a dose–response curve by nonlinear regression using GraphPad Prism 5.0 for curve fitting. For all data, standard error of the mean (SEM) values were ±<10%.

**Table 21:** Cytotoxicity of oxadiazoles **16a**–**i** against a panel of human cancer cell lines: lung (A549), breast (MDA-MB-231 and MCF-7), and cervical (HeLa).

Compound	R	R_1_	R_2_	IC_50_ (µM)			
				A549	MDA-MB-231	MCF-7	HeLa
**16a**	Br	CH_3_	H	- ^a^	-	30.9 ± 0.48	14.8 ± 0.39
**16b**	Br	CH_3_	Br	-	38.9 ± 0.85	24 ± 0.5	19.5 ± 0.43
**16c**	H	CH_3_	OCH_3_	-	-	32.2 ± 0.9	42.3 ± 0.96
**16d**	H	CH_3_	NO_2_	-	-	19.2 ± 0.8	33.2 ± 0.45
**16e**	Br	CH_3_	OCH_3_	24.2 ± 0.89	12.17 ± 1.1	1.8 ± 0.9	9.23 ± 0.58
**16f**	H	H	H	-	-	24.5 ± 0.9	9.4 ± 0.37
**16g**	H	H	Br	-	-	-	16.3 ± 0.33
**16h**	H	H	NO_2_	3.3 ± 0.85	10.23 ± 1.3	2.6 ± 0.89	6.34 ± 0.56
**16i**	Br	H	H	-	-	-	19.8 ± 0.45

^a^- no activity.

**Table 22:** Cytotoxicity of oxadiazoles **17a**,**b** against a panel of human cancer cell lines: breast (MCF-7), colon (HCT-116 and CaCo2), and cervical (HeLa).

Compound	R	R_1_	IC_50_ (µM) ^a^			
			MCF-7	HCT-116	CaCo2	HeLa
**17a**	Cl	Br	0.65 ± 0.05	1.93 ± 0.06	1.06 ± 0.09	10.56 ± 0.98
**17b**	OCH_3_	Br	2.41 ± 0.23	3.55 ± 0.1	3.33 ± 0.25	13.96 ± 1.41

^a^ IC_50_ value was calculated by plotting the percentage viability versus concentration on a logarithmic graph. Results are the mean values SD (standard deviation) of three separate experiments carried out in triplicate.

**Table 23:** *In vitro* inhibition of cancer cell lines growth in leukaemia, NSCLC, colon, CNS, melanoma, renal, and breast cancer subpanels by compounds **18a** and **b**.

Cell Line	GI_50_ (µM) ^a^		Cell Line	GI_50_ (µM) ^a^	
	18a	18b		18a	18b
Leukaemia			CNS Cancer		
CCRF-CEM	14.4	6.40	SF-268	16.0	5.29
HL-60(TB)	15.4	4.45	SF-295	17.1	2.52
K-562	42.1	2.78	SF-539	16.2	1.81
MOLT-4	4.75	1.55	SNB-19	27.0	4.09
RPMI82226	37.8	6.79	SNB-75	18.4	4.25
SR	3.46	2.36	U251	12.9	3.37
Non-small Cell Lung Cancer			Melanoma		
A549/ATCC	16.3	3.24	LOX IMVI	5.38	1.70
EKVX	27.2	3.15	MALME-3M	93.1	9.64
HOP-62	17.3	7.98	M14	>100	2.15
HOP-92	2.06	1.86	MDA-MB-435	ND ^b^	ND
NCI-H226	19.8	5.29	SK-MEL-2	57.5	2.34
NCI-H23	22.0	2.49	SK-MEL-28	>100	4.80
NCI-H322M	>100	5.73	SK-MEL-5	13.1	1.79
NCI-H460	4.48	2.35	UACC-257	80.6	4.27
NCI-H522	22.1	1.75	UACC-62	16.4	1.63
			Renal Cancer		
Colon Cancer			786-0	14.1	3.41
COLO 205	7.98	2.22	A498	19.6	2.44
HCC-2998	4.74	1.71	ACHN	7.67	3.23
HCT-116	19.0	3.82	CAKI-1	>100	1.70
HCT-15	8.68	3.01	RXF 393	14.7	3.03
HT29	5.35	3.57	SN12C	1.95	ND
KM12	4.58	3.45	TK-10	37.4	5.16
SW-620	>100	3.52	UO-31	37.6	3.01
			Breast Cancer		
			MCF-7	3.95	2.64
			NCI/ADR-RES	27.6	2.25
			MDA-MB-231/ATCC	8.06	2.95
			HS 578T	18.5	3.27
			BT-549	15.9	2.03
			T-47D	79.7	4.06
			MDA-MB-435	ND	2.99

^a^ Concentration (µM) that inhibits 50% net cell growth. ^b^ ND = Not Determined.

**Table 24:** Overview of the results of the *in vitro* anti-tumour screening for compounds **19**, **20a**, and **20b**.

Derivative	N° Cell Lines Tested	N° Active Cell Lines	GI_50_ Range (µM) ^a^
**19**	17	17	6.60–74.8
**20a**	17	16	2.47–15.5
**20b**	16	16	0.058–7.19

^a^ Concentration range that inhibits 50% net cell growth.

**Table 25:** *In vitro* inhibition of cancer cell lines growth in leukaemia, CNS, ovarian and breast cancer subpanels by compounds **21a**, **22a**–**d**.

Cell Line	GI_50_ (µM) ^a^				
	21a	22a	22b	22c	22d
Leukaemia					
CCRF-CEM	ND ^b^	ND	1.51	1.52	1.13
RPMI82226	2.74	0.37	1.91	3.90	2.80
SR	3.24	0.22	4.24	11.3	9.53
CNS Cancer					
SF-295	2.33	0.50	2.93	3.29	3.14
SF-539	4.21	0.16	4.37	11.7	7.14
SNB-19	1.15	0.56	4.81	7.88	5.26
U251	3.37	0.36	3.86	6.46	4.19
Ovarian Cancer					
IGROV1	3.24	0.25	<0.01	1.14	1.86
OVCAR-5	4.68	0.38	>100	5.50	8.13
Breast Cancer					
MCF-7	2.30	0.39	2.74	3.05	3.55
MDA-MB-435	3.31	0.22	4.52	5.87	5.76

^a^ Concentration (µM) that inhibits 50% net cell growth. ^b^ ND = Not Determined.

**Table 26:** Percentage of PMA-induced oedema inhibition in mouse ears by bis-indolyl compounds.

Compound	Dose	% Oedema Inhibition
Topsentin	50 µg/ear	70.6
Bromotopsentin	50 µg/ear	75.4
Topsentin monoacetate	50 µg/ear	45.8
Topsentin diacetate	50 µg/ear	42.6
Dragmacidin	50 µg/ear	64.0
Nortopsentin A	50 µg/ear	98.1
Nortopsentin B	50 µg/ear	38.2
Nortopsentin C	50 µg/ear	70.1
Hamacanthin A	50 µM	50.0
Hamacanthin B	50 µM	34.0

**Table 27:** The percentage of bee venom phospholipase A2 inactivation by bis-indolyl compounds.

Compound	Final Concentration	% Inactivation
Topsentin	1 µM	67
Bromotopsentin	1 µM	33
Topsentin monoacetate	1 µM	42
Topsentin diacetate	1 µM	32
Dragmacidin	1 µM	27
Nortopsentin A	1 µM	30
Nortopsentin B	1 µM	27
Nortopsentin C	1 µM	26
Hamacanthin A	1.6 µM	37
Hamacanthin B	1.6 µM	32

**Table 28:** RTX-induced oedema inhibition percentage by Nortopsentin C, Hamacanthin B, and Topsentin.

Dose µg/ear	% Oedema Inhibition		Dose µg/ear	% Oedema Inhibition
	Nortopsentin C	Hamacanthin B		Topsentin
50	98.4	96.9	50	82
25	90.2	81.6	25	41
12.5	86.8	86.8	10	31
6.25	45.1	46.0	5	12
3.12	5.9	68.0		

**Table 29:** ED_50_ values in oedema inhibition by Nortopsentin C, Hamacanthin B, and Topsentin.

	Nortopsentin C	Hamacanthin B	Topsentin
**ED_50_**	8 µg/ear	1.5 µg/ear	20 µg/ear

**Table 30:** Rat bNOS inhibition percentage by Hamacanthin A, Dragmacidin D, Nortopsentin C, Dragmacidin, and Topsentin at a concentration range of 1.0–50 µM.

Concentration µM	bNOS Inhibition Percentage %				
	Hamacanthin A	Dragmacidin D	Nortopsentin C	Dragmacidin	Topsentin
50	99.58	99.56	98.77	90.27	22.66
10	75.42	96.44	16.05	32.74	-0.39
5	43.33	74.22	9.88	3.54	5.47
1	35.42	16.89	2016	38.94	5.08

**Table 31:** *In vitro* GSK3β inhibitory activities of aminopyrazole derivatives **24** and **25**.

Compound	R	R_1_	IC_50_ (µM) ^a^
**24a**	5-F	CH_3_	1.28 ± 0.18
**24b**	5-Br	CH_3_	1.93 ± 0.22
**24c**	5-OCH_3_	CH_3_	1.76 ± 0.19
**25a**	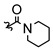	—	1.46 ± 0.04
**25b**	H	—	1.48 ± 0.30

^a^ The data represent mean values ± SEM of at least three independent experiments.

## Abbreviations

CNS	Central Nervous System
Ara-C	Cytarabine
FDA	Food and Drug Administration
NCI	National Cancer Institute
DMPM	Diffuse Malignant Peritoneal Mesothelioma
TVI	Tumour Volume Inhibition
CDK	Cyclin-Dependent Kinase
GSK3β	Glycogen synthase kinase 3β
CR-CSphCs	Colorectal Cancer Sphere Cells
MG_MID	Mean Graph Midpoint
bNOS	Neural nitric oxide synthase
LPS	Lipopolysaccharide
